# Correction: *Randomised controlled pilot feasibility trial of an early intervention programme for young infants with neurodevelopmental impairment in Uganda: a study protocol*

**DOI:** 10.1136/bmjopen-2019-032705corr1

**Published:** 2019-12-03

Nampijja M, Webb E, Nanyunja C, *et al*. Randomised controlled pilot feasibility trial of an early intervention programme for young infants with neurodevelopmental impairment in Uganda: a study protocol. *BMJ Open* 2019;9:e032705. doi: 10.1136/bmjopen-2019-032705.

This article was previously published with an error.

The first author of reference 2 was omitted. The correct reference is:

Olusanya BO, Davis AC, Wertlieb D, *et al*. Global Research on Developmental Disabilities Collaborators. Developmental disabilities among children younger than 5 years in 195 countries and territories, 1990–2016: a systematic analysis for the global burden of disease study 2016. *Lancet Glob Health* 2018;6:e1100–21.

