# Supplementary material for: Randomised controlled pilot feasibility trial of an early intervention programme for young infants with neurodevelopmental impairment in Uganda: a study protocol
Source: BMJ Open. 2019 Oct 9;9(10):e032705. doi: 10.1136/bmjopen-2019-032705 (PMC6797334; doi:10.1136/bmjopen-2019-032705)
Supplement: Author's manuscript [file bmjopen-2019-032705.draft_revisions.pdf]

BMJ Open is committed to open peer review. As part of this commitment we make the peer review history of every article we publish publicly available.

When an article is published we post the peer reviewers' comments and the authors' responses online. We also post the versions of the paper that were used during peer review. These are the versions that the peer review comments apply to.

The versions of the paper that follow are the versions that were submitted during the peer review process. They are not the versions of record or the final published versions. They should not be cited or distributed as the published version of this manuscript.

BMJ Open is an open access journal and the full, final, typeset and author-corrected version of record of the manuscript is available on our site with no access controls, subscription charges or pay-per-view fees (http://bmjopen.bmj.com).

If you have any questions on BMJ Open's open peer review process please email info.bmjopen@bmj.com

BMJ Open

A Randomised Controlled Pilot Feasibility Trial of an Early Intervention Programme for Young Infants with Neurodevelopmental Impairment in Uganda: Study Protocol

Journal:	*BMJ Open*
Manuscript ID	bmjopen-2019-032705
Article Type:	Protocol
Date Submitted by the Author:	03-Jul-2019
Complete List of Authors:	Nampijja, Margaret; MRC/UVRI Uganda Research Unit On AIDS; London School of Hygiene and Tropical Medicine<br>Webb, Emily; London School of Hygiene and Tropical Medicine Faculty of Epidemiology and Population Health<br>Nanyunja, Carol; MRC/UVRI Uganda Research Unit On AIDS<br>Sadoo, Samantha; London School of Hygiene and Tropical Medicine Faculty of Epidemiology and Population Health<br>Nalugya, Ruth; Medical Research Council / Uganda Virus Research Institute & London School of Hygiene & Tropical Medicine Uganda Research Unit<br>Nyonyintono, James; Kiwoko Hospital; Adara Development<br>Muhumuza, Anita; Mulago National Referral Hospital<br>Ssekidde, Moses; Kiwoko Hospital<br>Katumba, Kenneth; MRC/UVRI Uganda Research Unit On AIDS<br>Magnusson, Brooke; Adara Development, 300 Admiral Way, # 106, Edmonds<br>Kabugo, Daniel; Adara Development<br>Cowan, Frances; Imperial College London, Depart of Paediatrics<br>Martinez-Biarge, Miriam; Imperial College London, Depart of Paediatrics<br>Zuurmond, Maria; London School of Hygiene and Tropical Medicine Faculty of Infectious and Tropical Diseases<br>Morgan, Cathy; Cerebral Palsy Alliance<br>Lester, Deborah; Seattle Children's Hospital; Adara Development<br>Seeley, Janet; MRC/UVRI Uganda Research Unit On AIDS; London School of Hygiene and Tropical Medicine Faculty of Public Health and Policy<br>Tann, Cally; London School of Hygiene and Tropical Medicine Faculty of Epidemiology and Population Health; MRC/UVRI Uganda Research Unit On AIDS
Keywords:	Neonatal encephalopathy, Uganda, Neurodevelopmental impairment, Cohort study, Child disability, Developmental disability

SCHOLARONE™
Manuscripts

For peer review only - http://bmjopen.bmj.com/site/about/guidelines.xhtml

A Randomised Controlled Pilot Feasibility Trial of an Early Intervention Programme for Young Infants with Neurodevelopmental Impairment in Uganda: Study Protocol

Authors: Margaret Nampijja[1], Emily L Webb[2], Carol Nanyunja[1], Samantha Sadoo[3], Ruth Nalugya[1], James Nyonyintono[4,5], Anita Muhumuza[6], Moses Ssekidde[4], Kenneth R. Katumba[1], Brooke Magnusson[5], Daniel Kabugo[5], Frances M Cowan[7], Miriam Martinez-Biarge[7], Maria Zuurmond[8], Cathy Morgan[9,10], Debbie Lester [5,11], Janet Seeley[1,12], Cally Tann [1,3,13*]

Affiliations

[1]MRC/UVRI & LSHTM Uganda Research Unit, Plot 51-59 Nakiwogo Road, Entebbe, Uganda (Trial Sponsor)

[2]MRC Tropical Epidemiology Group, London School of Hygiene and Tropical Medicine, Keppel Street, London, UK

[3]Department of Infectious Disease Epidemiology, School of Hygiene and Tropical Medicine Keppel Street, London, UK

[4]Kiwoko Hospital, PO Box 149, Nakaseke, Uganda

[5]Adara Development, 300 Admiral Way, #106, Edmonds, Washington, USA

[6]Mulago National Referral Hospital, Makerere Hill Road, Kampala, Uganda

[7]Department of Paediatrics, Imperial College London, London, UK

[8]Department of Infectious & Tropical Diseases, London School of Hygiene and Tropical Medicine, Keppel Street, London, UK

[9]Paediatrics and Child Health, School of Medicine, University of Sydney, Sydney Australia

[10]Cerebral Palsy Alliance Research Institute, Sydney, Australia

[11]Seattle Children's Hospital, 4800 Sand Point Way NE, Seattle, Washington, USA

[12]Department of Global Health & Development, 15-17 Tavistock Place, London, UK

[13]Neonatal Medicine, University College London Hospitals NHS Trust, 235 Euston Rd, London, UK

*Corresponding author:

Dr Cally Tann

Email: cally.tann@lshtm.ac.uk

Tel: +44 (0) 7967 367 004

MARCH Centre, London School of Hygiene and Tropical Medicine, Keppel Street, London. WC1E 7HT

Key words: Neonatal encephalopathy, Uganda, outcomes, impairment, neurodevelopment, cohort study

For peer review only - http://bmjopen.bmj.com/site/about/guidelines.xhtml

ABSTRACT

Introduction:

Early intervention programmes for infants with neurodevelopmental impairment have been poorly studied especially in low-income settings. We aim to evaluate the feasibility and acceptability of a group participatory early intervention programme, the 'ABAaNA Early Intervention Programme' (EIP), for young children with neurodevelopmental impairment in Uganda.

Methods & Analysis:

We will conduct a pilot feasibility, single-blinded, randomised controlled trial comparing the EIP with standard care across two study sites (one urban, one rural) in central Uganda. Eligible infants (n=126, age 6-11 months) with neurodevelopmental impairment (defined as a developmental quotient <70 on Griffiths Scales of Mental Development, and, or Hammersmith Infant Neurological Examination score <60 will be recruited and randomised to the intervention or standard care arm. Intervention arm families will receive the 10-modular, peer-facilitated, participatory, community-based programme over 6 months. Recruited families will be followed up at 6 and 12 months after recruitment, and assessors will be blinded to the trial allocation. The primary hypothesis is that the ABAaNA EIP is feasible and acceptable when compared to standard care. Primary outcomes of interest are feasibility (number recruited and randomised at baseline) and acceptability (protocol violation of arm allocation and number of sessions attended) and family and child quality of life. Guided by the study aim, the qualitative data analysis will use a data-led thematic framework approach. The findings will inform scalability and sustainability of the programme.

Ethics & Dissemination: The trial protocol has been approved by the relevant Ugandan and UK ethics committees. Recruited families will give written informed consent and we will follow international codes for ethics and good clinical practice. Dissemination will be through peer-reviewed publications, conference presentations, and public engagement.

Trial Registration: ISRCTN44380971; protocol version 3.0, 19th February 2018

For peer review only

Article Summary

Strengths and limitations of this study

- This pilot feasibility trial is amongst the first to examine feasibility and acceptability of an early intervention programme for young children with neurodevelopmental impairment in a low resource sub-Saharan African setting.
- The mixed-methods evaluation of this complex community-level intervention will provide important information on implementation of an early intervention programme for child disability at scale.
- Whilst the small sample size and individually randomised trial design will limit our understanding of programme impact, quantitative and qualitative data will inform design and execution of a larger future trial to examine effects on important child and family outcomes.

For peer review only - http://bmjopen.bmj.com/site/about/guidelines.xhtml

INTRODUCTION

Globally each year, an estimated 30 million neonates experience complications around the time of birth which can have a life-long impact on health and development.(1) The United Nations Global Strategy for Women's, Children's and Adolescents' Health (2016-2030) advocates the need for all children not only to 'survive' but also to 'thrive'.(1) Whilst in recent decades substantial progress has been made in reducing child mortality in low- and middle-income countries (LMICs), the global burden of developmental disabilities remains unchanged.(2) Child neurodevelopmental impairment (NDI) significantly impacts families in any context, but particularly in low-resource settings, where availability and access to support services are limited, financial barriers greater, and social stigma more overt.(3)

A wide spectrum of impairment is seen after newborn illnesses, including cerebral palsy, ineffective feeding, learning, visual and hearing difficulties and epilepsy. A growing evidence base, largely from high-income countries (HICs), suggests that early intervention programmes (EIPs) commencing in the first months after birth, have the potential to limit and even prevent developmental and cognitive impairments following early brain injury. These programmes target the neuroplasticity of the immature developing brain, either directly or indirectly, through family capacity building and enrichment of the care-giving environment.(4)

In HICs, it has been shown that early environmental enrichment can enhance motor function in children <2 years.(5, 6) In LMICs, several trials have shown positive effects of EIPs in at-risk infants,(7-10) although these studies have not focused on infants specifically with NDI. Few studies have examined the feasibility and acceptability on affected children and their caregivers, and how they might be integrated into existing community health programmes.(11) Scalability and sustainability of an intervention programme is also dependent on its cost-effectiveness. This is particularly true in low- and middle-income countries where resources are scarce and existing care structures for children much less well established.

Aims

The study aims to evaluate whether a facilitated, community-based, participatory early intervention programme is feasible and acceptable. We will conduct a pilot feasibility single-blind, randomised-controlled trial (RCT) with two parallel groups. The outcomes of interest are feasibility of randomisation and recruitment, acceptability among caregivers and health care workers and early evidence of family impact quality of life, 6 months after recruitment and again 6 months later. The

For peer review only - http://bmjopen.bmj.com/site/about/guidelines.xhtml

incremental and protective cost-effectiveness of the EIP and the economic impact of child developmental disability to families and services in Uganda will be examined by Katumba K et al[1].

Objectives & Hypotheses

The primary objectives of the study are to:

1. Describe the feasibility and acceptability of the EIP for children with NDI and their families.
Hypothesis*: It will be feasible to conduct the EIP in rural and urban contexts and acceptable to families and the community.*

2. Obtain preliminary data on whether the EIP improves family quality of life when compared with SC.
Hypothesis: *Families receiving the community based EIP will demonstrate improved QoL scores on the Pediatric Quality of Life Family Impact module compared to SC 12 months after recruitment.*

3. Identify the main barriers and facilitating factors for scaling up the EIP.
Hypothesis*: The EIP is scalable in this low-resource Ugandan setting.*

4. Determine the incremental and protective cost-effectiveness of the ABAaNA EIP.
Hypothesis: *The ABAaNA EIP is a cost-effective intervention to improve family QoL for children with NDI.*

METHODS

We will conduct a pilot feasibility, single-blind, RCT with two parallel groups; one receiving the EIP and the other standard care.

Study Setting

The study is based at two Ugandan sites; one urban (Mulago Hospital, Kampala) and one rural (Kiwoko Hospital, Nakaseke). Neither site has existing family support services for children with NDI.

Mulago Hospital is the largest in Kampala, Uganda's capital city, taking high-risk pregnancies from across surrounding areas. Children's services include acute admissions, an inpatient malnutrition unit, and outpatients, with a weekly paediatric neurology clinic providing investigation and management of neurological conditions including seizures, and a clinic-based physiotherapy and occupational therapy service for children with cerebral palsy and other NDIs.

[1] Paired protocol paper of the economic analysis has been submitted to BMJ Open

For peer review only - http://bmjopen.bmj.com/site/about/guidelines.xhtml

Kiwoko Hospital in Nakaseke District, central Uganda, serves a catchment area of 800,000 people and provides comprehensive medical services, including neonatal inpatient care for >1200 infants per year. The trial implementation partner, Adara Development, has worked in partnership with Kiwoko Hospital since 1998, and the government to improve neonatal health in Nakaseke district. Together they provide HIV services, maternal health services, and community-based healthcare to 44 villages surrounding Kiwoko Hospital.

Participants and recruitment

Participants will be young children with NDI and their caregivers. A SPIRIT diagram showing the flow of participants is presented in Figure 1.

Figure 1: Flow of participants

Screening for eligibility

Infants at high-risk of NDI will be identified from i) neonatal admission registers and neonatal follow-up services, ii) local paediatric outpatient services iii) attendance for early child health services following community sensitisation. Sensitisation will include public health announcements on local radio raising awareness of the research and appropriate child development more generally. Caregivers of high-risk infants (survivors of neonatal encephalopathy (NE), prematurity, neonatal septicaemias/meningitis and severe jaundice) will be contacted by phone and invited to attend an appointment when the child is 6-11 months old. After informed written consent, they will be screened for NDI using the Malawi Developmental Assessment Tool (MDAT).(12) If two or more items in any MDAT domain are not achieved, the child will be referred for comprehensive neurodevelopmental assessment. If the child fails one item in two or more domains, they will be invited back for an assessment in one month. If the child's MDAT scores are age-appropriate across all domains, advice will be given on play and stimulation, communication, nutrition and immunisations and the child discharged.

Caregivers of infants screening positive on MDAT will be invited to an appointment for written informed consent, and comprehensive neurodevelopmental assessment by study staff using the Griffiths Mental Developmental Scales (GMDS)(13) and the Hammersmith Infant Neurological Examination (HINE)(14). An overall Developmental Quotient (DQ) will be derived, from the GMDS subscales assessing locomotor, personal-social, hearing/language, eye-hand co-ordination and performance skills.(13) Neuromotor impairment will be further assessed according to the HINE, a standardised paediatric neurological examination and classified by type. We have used both these

For peer review only - http://bmjopen.bmj.com/site/about/guidelines.xhtml
For peer review only

tests extensively in previous studies in Uganda and found them easy to administer in this setting and at this age.(15) The assessments will be conducted in the local language using the standard manual

Box 1: *Eligibility for inclusion in the RCT*

Inclusion Criteria:

- Infant aged 6-11 months
- Moderate-severe NDI defined as a GMDS DQ <70 and/or HINE score <60 (Romeo, 2013)
- Informed written consent by caregiver

Exclusion criteria:

- Infants aged >12 months
- Infants screening positive for NDI (using MDAT) but not meeting the criteria for moderate-severe NDI on GMDC & HINE assessment
- Conditions requiring prolonged inpatient treatment
- Parents unwilling or unable to attend the full programme
- Main residence outside Nakaseke or Luwero district, and >20km from Mulago Hospital

material to ensure internal consistency in assessments technique. Inclusion and exclusion criteria are outlined in Box 1.

Baseline characteristics

Infant and caregiver demographic information will be recorded at baseline. All outcome measures will also be measured at baseline enabling pre- and post-intervention comparisons.

Randomisation

Infants and their caregivers will be randomised in a 1:1 ratio to either the EIP or SC arm. Randomisation will be stratified by recruitment centre. Randomisation lists indicating a randomisation number and trial arm allocation, will be prepared by the trial statistician using a random number generator in Stata prior to the commencement of the study, and stored on a secure, password-protected computer at MRC/UVRI by a statistician otherwise not involved in the study. When a participant is eligible for recruitment and consent obtained, study staff will contact the MRC/UVRI statistician who will inform the study staff of the study number and trial arm to which the participant is to be allocated. The personnel in charge of the randomisation will not be involved in other study procedures, including assessment of outcomes.

For peer review only - http://bmjopen.bmj.com/site/about/guidelines.xhtml

Intervention arm

The early intervention programme is a community-based, peer-led group programme with caregivers at a community level, using a participatory approach driven by adult learning theory.(19) The programme manual is freely available to download (https://www.ubuntu-hub.org). Development of the programme is described in Box 2.

Box 2. Developing the ABAaNA early intervention programme

In LMICs, services for affected children are often lacking and parental levels of knowledge and understanding about cerebral palsy are often low. To fill this gap, a parent training programme called 'Getting to Know Cerebral Palsy' was developed and launched in partnership between the LSHTM and CBM (Christian Blind Mission) an international disability and development organisation (https://www.ubuntu-hub.org). The programme aims to increase parental knowledge and skills and promotes a participatory learning approach with an emphasis on the empowerment of caregivers across a broad spectrum of impairment for children aged 2-12 years.(16, 17)

Since 2011, the ABAaNA studies ('Abaana', meaning 'children in the local language Luganda) have been examining risk factors for, and outcomes from, neonatal encephalopathy (NE) in Uganda.(15) Studies examining early neurodevelopmental outcomes after NE revealed a high prevalence of NDI with 25% of those affected also having malnutrition from related feeding difficulties.(15) Qualitative work highlighted the stigma and broad-ranging social, emotional and financial impacts on affected families,(3) and the need for an intervention that may improve life chances amongst affected families.

The EIP was developed around the principles of 'Getting to Know Cerebral Palsy' (http://ubuntu-hub.org), and has been adapted for younger children aged 0-2 years following an iterative process following MRC recommendations on development and evaluation of complex interventions,(18); it was supported by a diverse Expert Advisory Group including local parents with children with NDI, Disabled Persons Organisations and experts in early intervention and child development. Core themes running through the programme are summarised in Figure 2. The newly developed programme was piloted amongst 28 families at Mulago Hospital in Kampala in 2015-6 and showed a 25% improvement in family quality of life scores (PedsQL, Family Impact module 2.0) post intervention (verbal communication).

Participating families are encouraged to share experiences through discussion and reflection, prioritise problems and identify solutions together. Facilitators of the group sessions are 'expert

parents', themselves parents of children with NDI, who have undergone five days of core training followed by regular supervision, face-to-face mentoring meetings and telephone discussions with existing in-country Master Facilitators (trained therapists in Uganda). Each EIP group involves 6-10 families; groups are selected depending on locality for ease of attendance. The training is divided into ten modules covering understanding disability, positioning and carrying, feeding, mobilising, communication, play, everyday activities, and experiences in the local community (Figure 2, Table 1). Individual module sessions are delivered every 1-2 weeks and last 2-3 hours including time for facilitated discussion; the entire programme is designed to be delivered over six months including at least one home visit conducted by the expert parent facilitator.

Figure 2: Core themes and content of the ABAaNA Early Intervention Programme

Table 1: Description of the programme modules

**Module**	**Content**
1. Let's get started	Content and ground rules of the programme<br>Understanding cerebral palsy, additional resources for information<br>Personal stories
2. Know your child	Developmental milestones for young children<br>Determining each child's progress
3. Positioning and carrying	The importance of optimal positioning<br>Practical skills regarding optimal positioning
4. Eating and drinking	Feeding challenges for children with neurodevelopmental impairment<br>Practical skills for addressing feeding challenges
5. Learning to move	Understanding different types of movement<br>Practical skills for assisting learning to move
6. Communicating	The importance of communication<br>Practical advice to encourage their child to communicate
7. Play and early stimulation	The importance of early stimulation and play for children to develop<br>Challenges of inclusion in play with the family and community<br>Creation of simple toys<br>How parents/ caregivers can encourage their child to play
8. Everyday activities	Using everyday activities to promote child development<br>Management of seizures<br>Review of previous sessions
9. Our community	Community resources available<br>Discussion around barriers to inclusion, addressing stigma and discrimination<br>Understanding disability rights<br>Thoughts and feelings of the caregiver<br>Members of community invited to attend this session
10. Next steps	Planning to facilitate their own group<br>Reflection on learning points<br>Endpoint data collection

For peer review only - http://bmjopen.bmj.com/site/about/guidelines.xhtml

Fidelity & Adherence to the intervention

EIP facilitators will receive a 5-day training programme delivered by two Master Facilitators, which includes facilitation skills, knowledge transfer on the core contents of the EIP manual and translation of knowledge to practice through simulated sessions with families and children with NDI. All trial intervention groups will be co-facilitated by a Master Facilitator providing supportive supervision to new facilitators. After each modular group meeting, a short-facilitated feedback session will be conducted, and the content of the module delivered will be recorded. Attendance of individual caregivers and children at the group sessions will be recorded. Facilitators will emphasize to caregivers the importance of attending all sessions, with phone calls prior to each session to promote adherence. If missed, a catch-up session may be offered before the next module.

Standard care arm

Standard care (SC) refers to care that is currently available in established local services. In both sites this includes referral to physiotherapy, seizure management and nutritional support. Information on access to local medical, therapy and family services will be collected. Families in the SC arm will be offered delayed entry into the EIP after completing the 18-month assessment.

Outcomes

Participants in both arms will be assessed by study staff masked to trial allocation at two time points; 6 months after recruitment which corresponds to completion of the EIP in the intervention arm (age 12-17 months), and again 6 months later (age 18-23 months) (Figure 2). Caregivers will be phoned a week before the follow-up assessments to arrange a time. Assessments will be primarily conducted in the study-site clinics. Where caregivers cannot be contacted by phone or are unable to attend the clinic, a community visit will be arranged and assessments completed at home. Outcome assessments will be conducted by Mulago assessors for children recruited at Kiwoko, and vice versa to ensure assessors are blind to allocation arm. Inter-rater reliability will be assessed.

Primary outcome measures

The primary outcomes of the study will be:

1. *Feasibility of participant recruitment and randomisation* as assessed by the total number recruited and randomised to each arm. Qualitative tools will also be used to capture information on feasibility.
2. *Acceptability of the EIP amongst caregivers and health care workers* as assessed by the protocol violation rate (e.g. participants in the intervention arm being treated as if they were in the control

For peer review only - http://bmjopen.bmj.com/site/about/guidelines.xhtml

arm or vice versa) at programme completion, and by the number of programme sessions attended between baseline and programme completion. Qualitative tools will also be used to capture information on acceptability.

3. *Preliminary evidence of impact on Family Quality of Life* as assessed using the scored Pediatric Quality of Life Family Impact module (PedsQL),(20) The PedsQL comprises 36 items scored on a 0-4 Likert scale and linearly transformed to a 0-100 scale, with higher scores indicating a better QoL. It will be translated into the local language Luganda and administered as a standardised structured interview by trained study staff.

Other outcomes of interest

1. *Child motor functioning* as assessed by the mobility score of the Pediatric Evaluation Disability Inventory (PEDI).(21) The PEDI is a standardised test designed to identify and describe functional impairment and monitor progress. Normative scaled scores are obtained for children ≥6 months to provide age-related expectations of ability.
2. *Child cognitive function* as assessed by the GMDS.(13)
3. *Child growth, health and well-being* assessed using weight, height and head circumference measured according to standardised protocol. Occipito-frontal head circumference (OFC, paper tape measure), weight, (SECA336 electronic scales, Hamburg, Germany) and height will be taken by study staff using standardised procedures. Haemoglobin will be determined on a finger prick sample using HemoCue Hb 201 (HemoCue AB, Angelholm, Sweden). A structured maternal interview in Luganda will report on caregivers concerns regarding health, growth and development of their child and episodes of illness including seizures and other neurological problems, feeding difficulties, chest infections, and treatment for malnutrition.
4. *Caregiver psychological distress* assessed using the Self-Referral Questionnaire (SRQ) and the Parenting Stress index (PSI).(22) The SRQ consists of 20 items each scored 0 (symptom absent) or 1 (symptom present) giving a total out of 20. The PSI is a 120-item inventory measuring the magnitude of caregiver stress attributable to parent-child relationship (Total Stress Scale), and situational/demographics factors outside the parent-child relationship (Life Stress Scale). These tools will be translated into Luganda.
5. *Caregiver-child attachment* assessed using the Maternal Infant Responsiveness Instrument (MIRI); a 22-item scale designed to measure the parent's feelings about their infant and an appraisal of the infant's responses.(23)
6. *Quality of the home environment* assessed using the Infant Toddler-Home Observation for the Measurement of the Environment (IT-HOME). This comprises 45 items, based on observation

For peer review only - http://bmjopen.bmj.com/site/about/guidelines.xhtml

and/or interview, assessing the physical environment of the home and the child's interaction within it.(24)

7. *Cost of illness and protective-effectiveness* will be assessed (Katumba K et al[2])

Qualitative methods

IDIs will be conducted with five randomly selected caregivers from each arm at each site. FGDs will be conducted with caregivers, at baseline, 6 months post-recruitment and again 6 months later in both the intervention and SC arms. Amongst intervention arm families, qualitative techniques will be used to capture information on the feasibility, acceptability and impact of the EIP intervention using qualitative tools including focus group discussions (FGDs), in-depth-interviews (IDIs) and observation.

We will describe the experiences of children and caregivers relating to the intervention received including the impact of the disability, parental confidence level, inclusion in community life and experience of stigma and discrimination. We will examine changes in these domains over the follow-up period and explore attributions of change. In addition, we will perform social mapping of parent networks and group discussions with staff on their perspectives and experiences of using the EIP. The interviews will be conducted by social scientists who have experience in qualitative research.

Data management and access

Data collected in the clinic or at field visits will be entered on standard clinical record forms (CRFs). Clinical data will be recorded under a unique study ID number. Completed CRFs will be checked by and double-entered into a trial-specific MS Access database. Data from both in-depth interviews (IDIs) and focus groups (FGDs) will be collected in the form of audiotapes, transcripts and field notes. All data entry and data management will be overseen by a statistician/data manager at the MRC/UVRI Unit. Data will be maintained on the host institution server and backed-up following standardised operating procedures. Paper CRFs will be stored in lockable filing cabinets at the sites. Access to these data during the trial will be restricted to essential personnel (the PIs, site co-investigators, medical research officers, and data clerks).

Confidentiality

All research-team members will receive training in confidentiality. Data will be stored without personal identifiers, except where names must be included to ensure identification of the correct

[2] Paired protocol paper of the economic analysis submitted to BMJ Open

For peer review only - http://bmjopen.bmj.com/site/about/guidelines.xhtml

participants for procedures. All data will be stored on password-protected computers, accessible only to research team members.

Sample size

The trial will recruit 126 children and their caregivers; 63 per arm. Allowing for a 20% dropout rate, this sample size will give 90% power to detect a minimal relative difference of 20% on PedQL Family Impact score between the intervention and control arms, at 5% significance level, assuming a mean PedQL score of 65 in the standard care arm and SD of 20 in both arms.

Statistical analysis

The first primary outcome, feasibility of participant recruitment and randomisation, will be assessed by the total number recruited and randomised to each arm. Recruitment and randomisation feasibility will be demonstrated if the target sample size of 126 is achieved. Data on participants screened, eligible and randomised will be displayed in a CONSORT flowchart. Descriptive statistics (frequencies, means, medians, standard deviations and interquartile ranges) will be used to describe the sample at baseline, by trial arm

The second primary outcome, acceptability, will be assessed quantitatively by (a) calculating the protocol violation rate and (b) summarising the number of programme sessions attended between baseline and programme completion for those in the intervention arm. Protocol violation rate will be calculated as the number of participants for whom a protocol violation occurs divided by the total number of participants, and will be presented both overall, and by trial arm. For participants in the EIP trial arm, the overall number of modules attended will be summarised using median, range and interquartile range. Acceptability on the basis of number of programme sessions will be defined as attendance of at least 6 modules.

For the third primary outcome and secondary outcomes, analyses will compare outcomes between intervention and control arms at the end of the programme, when the participants will be aged 12-17 months, and again 6 months later. Analysis will be on an intention-to-treat basis and missing data will not be imputed. Data for each outcome measure will be summarised by trial arm, using proportions for binary outcomes and means or medians for quantitative outcomes, depending on normality of the distribution. Differences in means/proportions between trial arms together with 95% confidence intervals will be calculated. We do not plan any formal statistical tests due to the preliminary nature of the trial; instead confidence intervals will provide a possible range of effect sizes. Regression models (linear regression for continuous outcomes, logistic regression for binary outcomes) will be used to adjust comparisons for baseline measures of the outcomes, which were

For peer review only - http://bmjopen.bmj.com/site/about/guidelines.xhtml

collected at enrolment into the trial, in order to improve precision of effectiveness estimates. For skewed continuous outcomes, data will be normalised before analysis using suitable transformations or quantile regression will be considered. No subgroup analyses are planned.

Qualitative data will be analysed using a thematic framework approach. Themes will be based on the study objectives and those emerging from the data. Social scientists (two people) will agree the coding frame and undertake analysis collaboratively to ensure agreement on the coding approach. Thematic summaries will be developed and shared with the wider team for discussion.

Trial management, data monitoring and reporting of adverse events

The TSC (25) will oversee progress of the study towards its objectives, review relevant information from other sources (e.g. other related trials) and receive reports from the Data and Safety Monitoring Board (DSMB). All adverse events, whether related to the intervention or not, will be noted and reported. A Data Monitoring and Safety Committee (DSMC) has been established independent of the investigators and the TSC but reporting to the TSC and the sponsor. The DSMB includes an expert on global child heath, a senior statistician, and a senior academic working in newborn and early child health research in Uganda, independent of the investigators. The DSMB will have access to all data on request. Resulting from the initial meeting of the DSMB on 28th June 2017, no formal stopping rules will be applied.

Children with NDI and particularly those with seizure disorders and difficulties with swallowing, are at increased mortality risk. All adverse events, whether related to the intervention or not, will be investigated and reported according to the UVRI Research Ethics Committee in accordance with GCP requirements. All deaths, hospitalisations and other serious adverse effects will be reported to the relevant ethics committee irrespective of whether the death or event is related to disease progression or not. Trial data monitoring will be conducted by an internal independent monitor at initiation, 6 months into data collection, again after one year and end of data collection.

Participant and public involvement

The intervention, study design and conduct, were developed directly from the engagement of caregivers and programme facilitators ('expert parents') with a parent representative on the TSC. The priorities and experiences of caregivers identified during facilitated group discussions at a key-stakeholders meeting (June 2017) contributed to the development of our research question and outcome measures. Plans to communicate findings to participants and the wider community will involve caregivers, through formal discussions with the TSC.

For peer review only - http://bmjopen.bmj.com/site/about/guidelines.xhtml

Ethics & Dissemination

Ethics

The protocol has been approved by the Research and Ethics committee of the UVRI, Mulago Hospital and Kiwoko Hospitals, the Uganda National Council for Science and Technology, the Uganda President's Office, and the ethics committee of the LSHTM. Information sheets will be available in English and Luganda, the main local language. Parents will be provided with an oral and written explanation of the study by Ugandan study staff to ensure that information is accessible to those with lower levels of literacy. Witnessed consent using a thumb print will be available to parents/guardians who are non-literate. Reimbursement for the cost of transport will be provided to caregivers on attendance at the screening and recruitment visits.

All recruited children will receive SC at the study sites. This will include referral to local services for seizure management and physiotherapy where available. To date the benefits of the proposed EIP have not been proven and may have a negative effect if children are incorrectly classified as having NDI and placed in the programme. Children and their caregivers in the control arm will receive delayed entry into the programme for older children ('Getting to Know Cerebral Palsy') at 18-23 months at the time of their final study assessments.

Dissemination

Our programme has strong links with partnership organisations working in Maternal and Child Health programming including Adara Development, Kiwoko Hospital, Nakaseke District Health Office and other collaborating institutions. Research findings will be disseminated to the Ministry of Health, to inform local and national health policies. Regional level stakeholders including the Nakaseke District Health Office and heads of regional health and social services, will be engaged to support staff recruitment, contributing to the sustainability of the innovation at local and district level. Meetings for key-stakeholders, including local NGOs working in child disability will be held twice during the project period to promote buy-in, facilitate fast-cycle learning, disseminate study findings and ultimately promote sustainability of the programme. Global learning will be facilitated through our existing online community-of-practice spanning 70 countries and >300 members. Communications support staff at the MRC/UVRI, LSHTM and Adara Development will facilitate dissemination of information through appropriate media outlets, the web and social media.

Study findings will be published through Open Access peer-reviewed journals, presentations at local, national and international conferences and to the local community through community meetings.

For peer review only - http://bmjopen.bmj.com/site/about/guidelines.xhtml

Written reports will be submitted to UVRI REC and reported to the trial registry. Data will be made available upon request.

Online supplementary information

1. Participant screening information and consent form
2. Participant recruitment information and consent form

Authors contributions

The study was conceived and designed by CT with substantial contribution from MN, DL, JN, EW, CM, JS, KK and FC. Research methodology was developed by MN, CN, MZ, JN, BM, DK, RN, MS, FC, MMB, AM and CT. The first version of the paper was written by CT & MN. All authors contributed to the final version of the manuscript.

Acknowledgements

The authors would like to thank the members of the ABAaNA Early Intervention Programme Expert Advisory Committee that supported the development of the programme and the implementation partners Kiwoko Hospital, Adara Development and Mulago University Hospital. The would like to acknowledge the contribution of Ellipsis for formatting the programme manual and thank Joanna Lawn for her contribution to manual illustrations. The authors would like to thank the funders, Grand Challenges Canada, Saving Brains and the Research Foundation of Cerebral Palsy Alliance Australia.

Funding statement

This work is supported by Saving Brains, Grand Challenges Canada grant number 1707-08687. The economic evaluation is supported by a Project Grant, awarded by the Research Foundation, Cerebral Palsy Alliance (PG02917).

The Trial sponsor is the MRC/UVRI and LSHTM Uganda Research Unit, Entebbe; Contact name: Prof Pontiano Kaleebu (Director); Address: MRC/UVRI & LSHTM Uganda Research Unit, Plot 51-59 Nakiwogo Road, P.O. Box 49 Entebbe, UGANDA Tel: +256 (0) 417 704000; mrc@mrcuganda.org

The funder had no role in the research design and will not have any role in the execution, analyses, interpretation of the data, or decision to submit results.

Data Statement

Data collected under this study will be protected according to the GDPR regulations.

For peer review only - http://bmjopen.bmj.com/site/about/guidelines.xhtml

Competing interest statement

We declare no competing interests.

References

1. WHO U. Survive and Thrive: Transforming Care for Every Small and Sick Newborn. Key Findings 2018 [Available from: https://www.unicef.org/every-child-alive/Survive-and-Thrive_KEY_FINDINGS_FINAL.pdf.

2. Olusanya BO, Davis AC, Wertlieb D, Boo N-Y, Nair MKC, Halpern R, et al. Developmental disabilities among children younger than 5 years in 195 countries and territories, 1990–2016: a systematic analysis for the Global Burden of Disease Study 2016. The Lancet Global Health. 2018;6(10):e1100-e21.

3. Nakamanya S, Siu GE, Lassman R, Seeley J, Tann CJ. Maternal experiences of caring for an infant with neurological impairment after neonatal encephalopathy in Uganda: a qualitative study. Disability and rehabilitation. 2015;37(16):1470-6.

4. Wallander JL, McClure E, Biasini F, Goudar SS, Pasha O, Chomba E, et al. Brain research to ameliorate impaired neurodevelopment--home-based intervention trial (BRAIN-HIT). BMC Pediatr. 2010;10:27.

5. Morgan C, Darrah J, Gordon AM, Harbourne R, Spittle A, Johnson R, et al. Effectiveness of motor interventions in infants with cerebral palsy: a systematic review. Developmental medicine and child neurology. 2016;58(9):900-9.

6. Palmer FB, Shapiro BK, Wachtel RC, Allen MC, Hiller JE, Harryman SE, et al. The effects of physical therapy on cerebral palsy. A controlled trial in infants with spastic diplegia. N Engl J Med. 1988;318(13):803-8.

7. Bao X, Sun S, Wei S. Early intervention promotes intellectual development of premature infants: a preliminary report. Early Intervention of Premature Infants Cooperative Research Group. Chin Med J (Engl). 1999;112(6):520-3.

8. Gardner JM, Walker SP, Powell CA, Grantham-McGregor S. A randomized controlled trial of a home-visiting intervention on cognition and behavior in term low birth weight infants. J Pediatr. 2003;143(5):634-9.

9. Walker SP, Chang SM, Powell CA, Grantham-McGregor SM. Psychosocial intervention improves the development of term low-birth-weight infants. J Nutr. 2004;134(6):1417-23.

10. Carlo WA, Goudar SS, Pasha O, Chomba E, Wallander JL, Biasini FJ, et al. Randomized trial of early developmental intervention on outcomes in children after birth asphyxia in developing countries. J Pediatr. 2013;162(4):705-12 e3.

11. Benfer KA, Novak I, Morgan C, Whittingham K, Khan NZ, Ware RS, et al. Community-based parent-delivered early detection and intervention programme for infants at high risk of cerebral palsy in a low-resource country (Learning through Everyday Activities with Parents (LEAP-CP): protocol for a randomised controlled trial. BMJ open. 2018;8(6):e021186.

12. Gladstone M, Lancaster GA, Umar E, Nyirenda M, Kayira E, van den Broek NR, et al. The Malawi Developmental Assessment Tool (MDAT): the creation, validation, and reliability of a tool to assess child development in rural African settings. PLoS Med. 2010;7(5):e1000273.

13. Griffiths R, Huntley M. The Griffiths mental development scales : from birth to two years : manual. Henley on Thames: ARICD; 1996.

14. Haataja L, Mercuri E, Regev R, Cowan F, Rutherford M, Dubowitz V, et al. Optimality score for the neurologic examination of the infant at 12 and 18 months of age. J Pediatr. 1999;135(2 Pt 1):153-61.

For peer review only - http://bmjopen.bmj.com/site/about/guidelines.xhtml

15. Tann CJ, Webb EL, Lassman R, Ssekyewa J, Sewegaba M, Musoke M, et al. Early Childhood Outcomes After Neonatal Encephalopathy in Uganda: A Cohort Study. EClinicalMedicine. 2018;6:26-35.
16. Zuurmond M, O'Banion D, Gladstone M, Carsamar S, Kerac M, Baltussen M, et al. Evaluating the impact of a community-based parent training programme for children with cerebral palsy in Ghana. PloS one. 2018;13(9):e0202096.
17. Zuurmond M, Nyante G, Baltussen M, Seeley J, Abanga J, Shakespeare T, et al. A support programme for caregivers of children with disabilities in Ghana: Understanding the impact on the wellbeing of caregivers. Child Care Health Dev. 2019;45(1):45-53.
18. Campbell M, Fitzpatrick R, Haines A, Kinmonth AL, Sandercock P, Spiegelhalter D, et al. Framework for design and evaluation of complex interventions to improve health. BMJ. 2000;321(7262):694-6.
19. Knowles M. The Adult Learning Theory - Andragogy 1984 [Available from: https://elearningindustry.com/the-adult-learning-theory-andragogy-of-malcolm-knowles.
20. Varni JW, Burwinkle TM, Seid M, Skarr D. The PedsQL 4.0 as a pediatric population health measure: feasibility, reliability, and validity. Ambul Pediatr. 2003;3(6):329-41.
21. Haley SM, Coster WJ, Ludlow LH, Haltiwanger JT, Andrellos PJ. Pediatric Evaluation of Disability Inventory. Pediatric Physical Therapy Pediatric Physical Therapy. 1994;6(1):42.
22. Abidin RR. Parenting Stress Index. Lutz, FL: PAR; 2012.
23. Amankwaa L. The Maternal Infant Responsiveness Instrument 2002 [Available from: https://miriinstrument.wordpress.com/.
24. Caldwell BM, Bradley RH, University of Arkansas at Little R, Center for Child D, Education. Administration manual : HOME observation for measurement of the environment. Little Rock, Ark.: University of Arkansas at Little Rock; 1984.
25. MRC. Guidelines for good clinical practice in clinical trials 1998 [Available from: https://mrc.ukri.org/documents/pdf/good-clinical-practice-in-clinical-trials/.

For peer review only

For peer review only - http://bmjopen.bmj.com/site/about/guidelines.xhtml

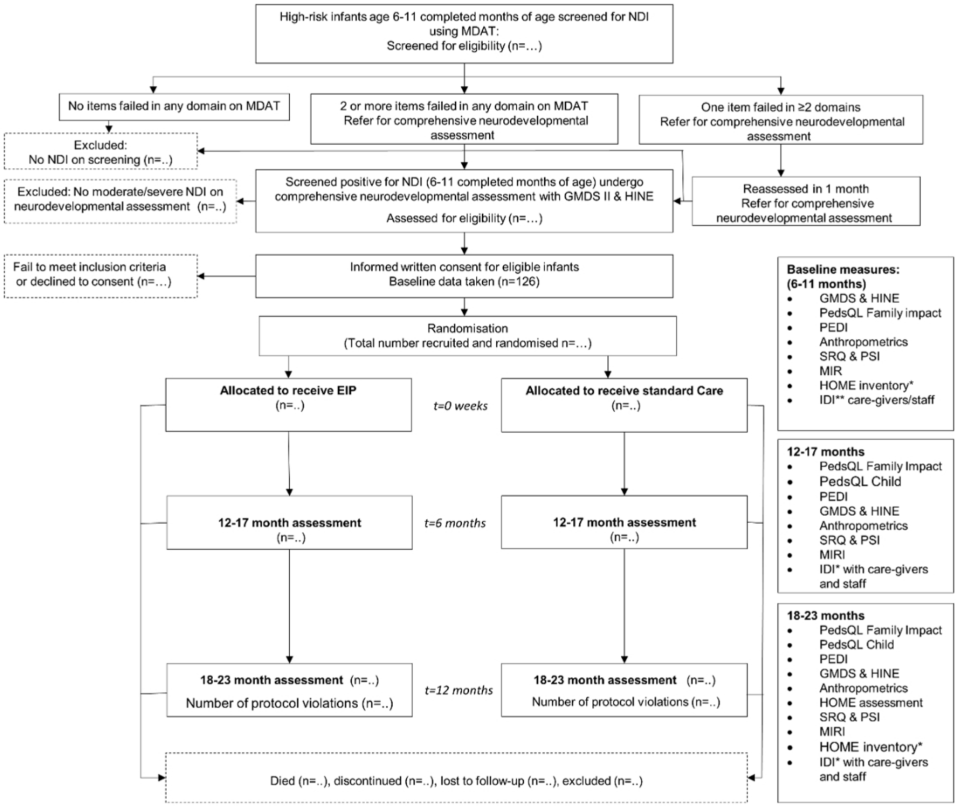

*In-depth interviews (IDI) with caregivers on impact of disability, confidence level of the parents, level of participation in family and community life and experience of stigma/discrimination. MDAT=Malawi Developmental Assessment Tool, PedQL=Pediatric Quality of Life tool, PEDI=Pediatric Evaluation Disability Inventory GMDS=Griffiths Mental Developmental Scales, HINE=Hammersmith Infant Neurological Examination, HOME=Home Observation for the Measurement of the Environment, SRQ=Self-Referral Questionnaire, PSI=Parent Stress Index MIRI=Maternal Infant Responsiveness Inventory

Figure 1: Flow of participants

303x345mm (300 x 300 DPI)

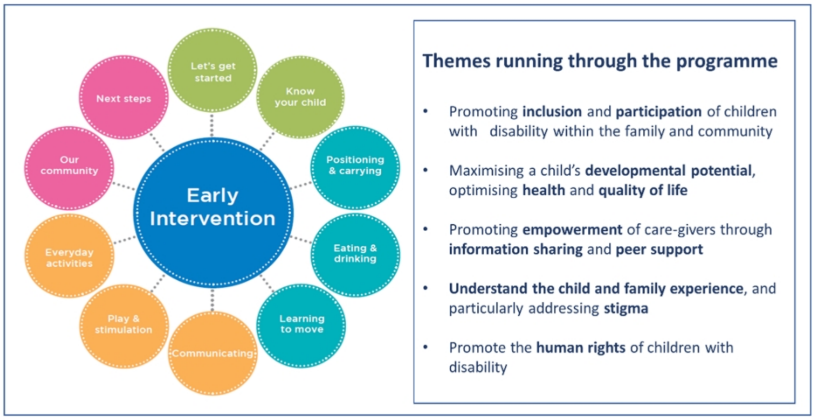

Figure 2: Core themes and content of the ABAaNA Early Intervention Programme

338x190mm (300 x 300 DPI)

SPIRIT

STANDARD PROTOCOL ITEMS: RECOMMENDATIONS FOR INTERVENTIONAL TRIALS

SPIRIT 2013 Checklist: Recommended items to address in a clinical trial protocol and related documents*

Section/item	Item No	Description	Addressed on page number
**Administrative information**			
Title	1	Descriptive title identifying the study design, population, interventions, and, if applicable, trial acronym	1
Trial registration	2a	Trial identifier and registry name. If not yet registered, name of intended registry	2
	2b	All items from the World Health Organization Trial Registration Data Set	N/A
Protocol version	3	Date and version identifier	2
Funding	4	Sources and types of financial, material, and other support	16
Roles and responsibilities	5a	Names, affiliations, and roles of protocol contributors	1 &
	5b	Name and contact information for the trial sponsor	1
	5c	Role of study sponsor and funders, if any, in study design; collection, management, analysis, and interpretation of data; writing of the report; and the decision to submit the report for publication, including whether they will have ultimate authority over any of these activities	16
	5d	Composition, roles, and responsibilities of the coordinating centre, steering committee, endpoint adjudication committee, data management team, and other individuals or groups overseeing the trial, if applicable (see Item 21a for data monitoring committee)	13/14

For peer review only

Introduction

Background and rationale	6a	Description of research question and justification for undertaking the trial, including summary of relevant studies (published and unpublished) examining benefits and harms for each intervention	______4______
	6b	Explanation for choice of comparators	______4___
Objectives	7	Specific objectives or hypotheses	______5______
Trial design	8	Description of trial design including type of trial (eg, parallel group, crossover, factorial, single group), allocation ratio, and framework (eg, superiority, equivalence, noninferiority, exploratory)	_____4-5______

Methods: Participants, interventions, and outcomes

Study setting	9	Description of study settings (eg, community clinic, academic hospital) and list of countries where data will be collected. Reference to where list of study sites can be obtained	_____5-6_____
Eligibility criteria	10	Inclusion and exclusion criteria for participants. If applicable, eligibility criteria for study centres and individuals who will perform the interventions (eg, surgeons, psychotherapists)	_____7 (Box 1)_
Interventions	11a	Interventions for each group with sufficient detail to allow replication, including how and when they will be administered	____8-10_____
	11b	Criteria for discontinuing or modifying allocated interventions for a given trial participant (eg, drug dose change in response to harms, participant request, or improving/worsening disease)	____N/A_____
	11c	Strategies to improve adherence to intervention protocols, and any procedures for monitoring adherence (eg, drug tablet return, laboratory tests)	____10_______
	11d	Relevant concomitant care and interventions that are permitted or prohibited during the trial	____10_______
Outcomes	12	Primary, secondary, and other outcomes, including the specific measurement variable (eg, systolic blood pressure), analysis metric (eg, change from baseline, final value, time to event), method of aggregation (eg, median, proportion), and time point for each outcome. Explanation of the clinical relevance of chosen efficacy and harm outcomes is strongly recommended	____10-11____
Participant timeline	13	Time schedule of enrolment, interventions (including any run-ins and washouts), assessments, and visits for participants. A schematic diagram is highly recommended (see Figure)	__10 &Figure 2

For peer review only - http://bmjopen.bmj.com/site/about/guidelines.xhtml

Sample size	14	Estimated number of participants needed to achieve study objectives and how it was determined, including clinical and statistical assumptions supporting any sample size calculations	___12-13_____
Recruitment	15	Strategies for achieving adequate participant enrolment to reach target sample size	___6-7______

Methods: Assignment of interventions (for controlled trials)

Allocation:

Sequence generation	16a	Method of generating the allocation sequence (eg, computer-generated random numbers), and list of any factors for stratification. To reduce predictability of a random sequence, details of any planned restriction (eg, blocking) should be provided in a separate document that is unavailable to those who enrol participants or assign interventions	____7-8______
Allocation concealment mechanism	16b	Mechanism of implementing the allocation sequence (eg, central telephone; sequentially numbered, opaque, sealed envelopes), describing any steps to conceal the sequence until interventions are assigned	____7-8_____
Implementation	16c	Who will generate the allocation sequence, who will enrol participants, and who will assign participants to interventions	_____7-8______
Blinding (masking)	17a	Who will be blinded after assignment to interventions (eg, trial participants, care providers, outcome assessors, data analysts), and how	___2,5,10______
	17b	If blinded, circumstances under which unblinding is permissible, and procedure for revealing a participant's allocated intervention during the trial	____13-14______

Methods: Data collection, management, and analysis

Data collection methods	18a	Plans for assessment and collection of outcome, baseline, and other trial data, including any related processes to promote data quality (eg, duplicate measurements, training of assessors) and a description of study instruments (eg, questionnaires, laboratory tests) along with their reliability and validity, if known. Reference to where data collection forms can be found, if not in the protocol	____6-11______
	18b	Plans to promote participant retention and complete follow-up, including list of any outcome data to be collected for participants who discontinue or deviate from intervention protocols	____10______

Data management	19	Plans for data entry, coding, security, and storage, including any related processes to promote data quality (eg, double data entry; range checks for data values). Reference to where details of data management procedures can be found, if not in the protocol	_____12______
Statistical methods	20a	Statistical methods for analysing primary and secondary outcomes. Reference to where other details of the statistical analysis plan can be found, if not in the protocol	_____13_______
	20b	Methods for any additional analyses (eg, subgroup and adjusted analyses)	_______N/A_____
	20c	Definition of analysis population relating to protocol non-adherence (eg, as randomised analysis), and any statistical methods to handle missing data (eg, multiple imputation)	_______13_______
**Methods: Monitoring**			
Data monitoring	21a	Composition of data monitoring committee (DMC); summary of its role and reporting structure; statement of whether it is independent from the sponsor and competing interests; and reference to where further details about its charter can be found, if not in the protocol. Alternatively, an explanation of why a DMC is not needed	_____13-14____
	21b	Description of any interim analyses and stopping guidelines, including who will have access to these interim results and make the final decision to terminate the trial	____13-14_____
Harms	22	Plans for collecting, assessing, reporting, and managing solicited and spontaneously reported adverse events and other unintended effects of trial interventions or trial conduct	_____14______
Auditing	23	Frequency and procedures for auditing trial conduct, if any, and whether the process will be independent from investigators and the sponsor	____14_______
**Ethics and dissemination**			
Research ethics approval	24	Plans for seeking research ethics committee/institutional review board (REC/IRB) approval	____14_______
Protocol amendments	25	Plans for communicating important protocol modifications (eg, changes to eligibility criteria, outcomes, analyses) to relevant parties (eg, investigators, REC/IRBs, trial participants, trial registries, journals, regulators)	_____15______

Consent or assent	26a	Who will obtain informed consent or assent from potential trial participants or authorised surrogates, and how (see Item 32)	_____14_______
	26b	Additional consent provisions for collection and use of participant data and biological specimens in ancillary studies, if applicable	_____N/A________
Confidentiality	27	How personal information about potential and enrolled participants will be collected, shared, and maintained in order to protect confidentiality before, during, and after the trial	_____12________
Declaration of interests	28	Financial and other competing interests for principal investigators for the overall trial and each study site	_____16________
Access to data	29	Statement of who will have access to the final trial dataset, and disclosure of contractual agreements that limit such access for investigators	_____15________
Ancillary and post-trial care	30	Provisions, if any, for ancillary and post-trial care, and for compensation to those who suffer harm from trial participation	______14-15____
Dissemination policy	31a	Plans for investigators and sponsor to communicate trial results to participants, healthcare professionals, the public, and other relevant groups (eg, via publication, reporting in results databases, or other data sharing arrangements), including any publication restrictions	_____15________
	31b	Authorship eligibility guidelines and any intended use of professional writers	_____16________
	31c	Plans, if any, for granting public access to the full protocol, participant-level dataset, and statistical code	____15_________
**Appendices**			
Informed consent materials	32	Model consent form and other related documentation given to participants and authorised surrogates	__online______
Biological specimens	33	Plans for collection, laboratory evaluation, and storage of biological specimens for genetic or molecular analysis in the current trial and for future use in ancillary studies, if applicable	_____N/A________

*It is strongly recommended that this checklist be read in conjunction with the SPIRIT 2013 Explanation & Elaboration for important clarification on the items. Amendments to the protocol should be tracked and dated. The SPIRIT checklist is copyrighted by the SPIRIT Group under the Creative Commons "Attribution-NonCommercial-NoDerivs 3.0 Unported" license.

For peer review only - http://bmjopen.bmj.com/site/about/guidelines.xhtml

BMJ Open

A Randomised Controlled Pilot Feasibility Trial of an Early Intervention Programme for Young Infants with Neurodevelopmental Impairment in Uganda: Study Protocol

Journal:	*BMJ Open*
Manuscript ID	bmjopen-2019-032705.R1
Article Type:	Protocol
Date Submitted by the Author:	13-Aug-2019

Complete List of Authors:	Nampijja, Margaret; MRC/UVRI Uganda Research Unit On AIDS; London School of Hygiene and Tropical Medicine<br>Webb, Emily; London School of Hygiene and Tropical Medicine Faculty of Epidemiology and Population Health<br>Nanyunja, Carol; MRC/UVRI Uganda Research Unit On AIDS<br>Sadoo, Samantha; London School of Hygiene and Tropical Medicine Faculty of Epidemiology and Population Health<br>Nalugya, Ruth; Medical Research Council / Uganda Virus Research Institute & London School of Hygiene & Tropical Medicine Uganda Research Unit<br>Nyonyintono, James; Kiwoko Hospital; Adara Development<br>Muhumuza, Anita; Mulago National Referral Hospital<br>Ssekidde, Moses; Kiwoko Hospital<br>Katumba, Kenneth; MRC/UVRI Uganda Research Unit On AIDS<br>Magnusson, Brooke; Adara Development, 300 Admiral Way, # 106, Edmonds<br>Kabugo, Daniel; Adara Development<br>Cowan, Frances; Imperial College London, Depart of Paediatrics<br>Martinez-Biarge, Miriam; Imperial College London, Depart of Paediatrics<br>Zuurmond, Maria; London School of Hygiene and Tropical Medicine Faculty of Infectious and Tropical Diseases<br>Morgan, Cathy; Cerebral Palsy Alliance<br>Lester, Deborah; Seattle Children's Hospital; Adara Development<br>Seeley, Janet; MRC/UVRI Uganda Research Unit On AIDS; London School of Hygiene and Tropical Medicine Faculty of Public Health and Policy<br>Tann, Cally; London School of Hygiene and Tropical Medicine Faculty of Epidemiology and Population Health; MRC/UVRI Uganda Research Unit On AIDS

**Primary Subject Heading**:	Paediatrics
Secondary Subject Heading:	Global health, Qualitative research, Rehabilitation medicine
Keywords:	Neonatal encephalopathy, Uganda, Neurodevelopmental impairment, Cohort study, Child disability, Developmental disability

For peer review only - http://bmjopen.bmj.com/site/about/guidelines.xhtml

A Randomised Controlled Pilot Feasibility Trial of an Early Intervention Programme for Young Infants with Neurodevelopmental Impairment in Uganda: Study Protocol

Authors: Margaret Nampijja[1], Emily L Webb[2], Carol Nanyunja[1], Samantha Sadoo[3], Ruth Nalugya[1], James Nyonyintono[4,5], Anita Muhumuza[6], Moses Ssekidde[4], Kenneth R. Katumba[1], Brooke Magnusson[5], Daniel Kabugo[5], Frances M Cowan[7], Miriam Martinez-Biarge[7], Maria Zuurmond[8], Cathy Morgan[9,10], Debbie Lester [5,11], Janet Seeley[1,12], Cally Tann [1,3,13*]

Affiliations

[1]MRC/UVRI & LSHTM Uganda Research Unit, Plot 51-59 Nakiwogo Road, Entebbe, Uganda (Trial Sponsor)

[2]MRC Tropical Epidemiology Group, London School of Hygiene and Tropical Medicine, Keppel Street, London, UK

[3]Department of Infectious Disease Epidemiology, School of Hygiene and Tropical Medicine, Keppel Street, London, UK

[4]Kiwoko Hospital, PO Box 149, Nakaseke, Uganda

[5]Adara Development, 300 Admiral Way, #106, Edmonds, Washington, USA

[6]Mulago National Referral Hospital, Makerere Hill Road, Kampala, Uganda

[7]Department of Paediatrics, Imperial College London, London, UK

[8]Department of Infectious & Tropical Diseases, London School of Hygiene and Tropical Medicine, Keppel Street, London, UK

[9]Paediatrics and Child Health, School of Medicine, University of Sydney, Sydney Australia

[10]Cerebral Palsy Alliance Research Institute, Sydney, Australia

[11]Seattle Children's Hospital, 4800 Sand Point Way NE, Seattle, Washington, USA

[12]Department of Global Health & Development, London School of Hygiene & Tropical Medicine, 15-17 Tavistock Place, London, UK

[13]Neonatal Medicine, University College London Hospitals NHS Trust, 235 Euston Rd, London, UK

*Corresponding author:

Dr Cally Tann

Email: cally.tann@lshtm.ac.uk

Tel: +44 (0) 7967 367 004

MARCH Centre, London School of Hygiene and Tropical Medicine, Keppel Street, London. WC1E 7HT

Key words: Neonatal encephalopathy, Uganda, outcomes, impairment, neurodevelopment, cohort study

For peer review only
For peer review only - http://bmjopen.bmj.com/site/about/guidelines.xhtml

ABSTRACT

Introduction:

Early intervention programmes for infants with neurodevelopmental impairment have been poorly studied especially in low-income settings. We aim to evaluate the feasibility and acceptability of a group participatory early intervention programme, the 'ABAaNA Early Intervention Programme' (EIP), for young children with neurodevelopmental impairment in Uganda.

Methods & Analysis:

We will conduct a pilot feasibility, single-blinded, randomised controlled trial comparing the EIP with standard care across two study sites (one urban, one rural) in central Uganda. Eligible infants (n=126, age 6-11 months) with neurodevelopmental impairment (defined as a developmental quotient <70 on Griffiths Scales of Mental Development, and, or Hammersmith Infant Neurological Examination score <60 will be recruited and randomised to the intervention or standard care arm. Intervention arm families will receive the 10-modular, peer-facilitated, participatory, community-based programme over 6 months. Recruited families will be followed up at 6 and 12 months after recruitment, and assessors will be blinded to the trial allocation. The primary hypothesis is that the ABAaNA EIP is feasible and acceptable when compared to standard care. Primary outcomes of interest are feasibility (number recruited and randomised at baseline) and acceptability (protocol violation of arm allocation and number of sessions attended) and family and child quality of life. Guided by the study aim, the qualitative data analysis will use a data-led thematic framework approach. The findings will inform scalability and sustainability of the programme.

Ethics & Dissemination: The trial protocol has been approved by the relevant Ugandan and UK ethics committees. Recruited families will give written informed consent and we will follow international codes for ethics and good clinical practice. Dissemination will be through peer-reviewed publications, conference presentations, and public engagement.

Trial Registration: ISRCTN44380971; protocol version 3.0, 19th February 2018

For peer review only - http://bmjopen.bmj.com/site/about/guidelines.xhtml

Article Summary

Strengths and limitations of this study

- This pilot feasibility trial is amongst the first to examine feasibility and acceptability of an early intervention programme for young children with neurodevelopmental impairment in a low resource sub-Saharan African setting.
- The mixed-methods evaluation of this complex community-level intervention will provide important information on implementation of an early intervention programme for child disability at scale.
- Whilst the small sample size and individually randomised trial design will limit our understanding of programme impact, quantitative and qualitative data will inform design and execution of a larger future trial to examine effects on important child and family outcomes.

For peer review only

For peer review only - http://bmjopen.bmj.com/site/about/guidelines.xhtml

INTRODUCTION

Globally each year, an estimated 30 million neonates experience complications around the time of birth which can have a life-long impact on health and development.(1) The United Nations Global Strategy for Women's, Children's and Adolescents' Health (2016-2030) advocates the need for all children not only to 'survive' but also to 'thrive'.(1) Whilst in recent decades substantial progress has been made in reducing child mortality in low- and middle-income countries (LMICs), the global burden of developmental disabilities remains unchanged.(2) Child neurodevelopmental impairment (NDI) significantly impacts families in any context, but particularly in low-resource settings, where availability and access to support services are limited, financial barriers greater, and social stigma more overt.(3)

A wide spectrum of impairment is seen after newborn illnesses, including cerebral palsy, ineffective feeding, learning, visual and hearing difficulties and epilepsy. A growing evidence base, largely from high-income countries (HICs), suggests that early intervention programmes (EIPs) commencing in the first months after birth, have the potential to limit and even prevent developmental and cognitive impairments following early brain injury. These programmes target the neuroplasticity of the immature developing brain, either directly or indirectly, through family capacity building and enrichment of the care-giving environment.(4)

In HICs, it has been shown that early environmental enrichment can enhance motor function in children <2 years.(5, 6) In LMICs, several trials have shown positive effects of EIPs in at-risk infants,(7-10) although these studies have not focused on infants specifically with NDI. Few studies have examined the feasibility and acceptability on affected children and their caregivers, and how they might be integrated into existing community health programmes.(11) Scalability and sustainability of an intervention programme is also dependent on its cost-effectiveness. This is particularly true in low- and middle-income countries where resources are scarce and existing care structures for children much less well established.

Aims

The study aims to evaluate whether a facilitated, community-based, participatory early intervention programme is feasible and acceptable. We will conduct a pilot feasibility single-blind, randomised-controlled trial (RCT) with two parallel groups. The outcomes of interest are feasibility of randomisation and recruitment, acceptability among caregivers and health care workers and early evidence of family impact quality of life, 6 months after recruitment and again 6 months later. The incremental and protective cost-effectiveness of the EIP and the economic impact of child

For peer review only - http://bmjopen.bmj.com/site/about/guidelines.xhtml

For peer review only

developmental disability to families and services in Uganda will be examined by Katumba et al in a separate protocol.

Objectives & Hypotheses

The primary objectives of the study are to:

1. Describe the feasibility and acceptability of the EIP for children with NDI and their families.
Hypothesis: *It will be feasible to conduct an RCT of the EIP versus standard care (SC) in rural and urban contexts and acceptable to families and the community.*

2. Obtain preliminary data on whether the EIP improves family quality of life when compared with SC.
Hypothesis: *Families receiving the community based EIP will demonstrate improved QoL scores on the Paediatric Quality of Life Family Impact module compared to SC 12 months after recruitment.*

3. Identify the main barriers and facilitating factors for scaling up the EIP.
Hypothesis: *The EIP is scalable in this low-resource Ugandan setting.*

4. Determine the incremental and protective cost-effectiveness of the ABAaNA EIP.
Hypothesis: *The ABAaNA EIP is a cost-effective intervention to improve family QoL for children with NDI.*

METHODS

We will conduct a pilot feasibility, single-blind, RCT with two parallel groups; one receiving the EIP and the other SC.

Study Setting

The study is based at two Ugandan sites; one urban (Mulago Hospital, Kampala) and one rural (Kiwoko Hospital, Nakaseke). Neither site has existing family support services for children with NDI.

Mulago Hospital is the largest in Kampala, Uganda's capital city, taking high-risk pregnancies from across surrounding areas. Children's services include acute admissions, an inpatient malnutrition unit, and outpatients, with a weekly paediatric neurology clinic providing investigation and management of neurological conditions including seizures, and a clinic-based physiotherapy and occupational therapy service for children with cerebral palsy and other NDIs.

For peer review only - http://bmjopen.bmj.com/site/about/guidelines.xhtml

Kiwoko Hospital in Nakaseke District, central Uganda, serves a catchment area of 800,000 people and provides comprehensive medical services, including neonatal inpatient care for >1200 infants per year. The trial implementation partner, Adara Development, has worked in partnership with Kiwoko Hospital since 1998, and the government to improve neonatal health in Nakaseke district. Together they provide HIV services, maternal health services, and community-based healthcare to 44 villages surrounding Kiwoko Hospital.

Participants and recruitment

Participants will be young children with NDI and their caregivers. A SPIRIT diagram showing the planned flow of participants is presented in Figure 1.

Figure 1: Flow of participants

Screening for eligibility

Infants at high-risk of NDI will be identified from i) neonatal admission registers and neonatal follow-up services, ii) local paediatric outpatient services iii) attendance for early child health services following community sensitisation. Sensitisation will include public health announcements on local radio raising awareness of the research and appropriate child development more generally. Caregivers of high-risk infants (survivors of neonatal encephalopathy (NE), prematurity, neonatal septicaemias/meningitis and severe jaundice) will be contacted by phone and invited to attend an appointment when the child is 6-11 months old. After informed written consent, they will be screened for NDI by trained study staff using the Malawi Developmental Assessment Tool (MDAT).(12) If two or more items in any MDAT domain are not achieved, the child will be referred for comprehensive neurodevelopmental assessment. If the child fails one item in two or more domains, they will be invited back for an assessment in one month. If the child's MDAT scores are age-appropriate across all domains, advice will be given on play and stimulation, communication, nutrition and immunisations and the child discharged.

Caregivers of infants screening positive on MDAT will be invited to an appointment for written informed consent, and if provided, comprehensive neurodevelopmental assessment by study staff using the Griffiths Mental Developmental Scales (GMDS)(13) and the Hammersmith Infant Neurological Examination (HINE)(14). An overall Developmental Quotient (DQ) will be derived, from the GMDS subscales assessing locomotor, personal-social, hearing/language, eye-hand co-ordination and performance skills.(13) Neuromotor impairment will be further assessed according to the HINE, a standardised paediatric neurological examination and classified by type. We have used both these

For peer review only - http://bmjopen.bmj.com/site/about/guidelines.xhtml

tests extensively in previous studies in Uganda and found them easy to administer in this setting and at this age.(15) The assessments will be conducted in the local language using the standard manual

Box 1: *Eligibility for inclusion in the RCT*

Inclusion Criteria:

- Infant aged 6-11 months
- Moderate-severe NDI defined as a GMDS DQ <70 and/or HINE score <60 (Romeo, 2013)
- Informed written consent by caregiver

Exclusion criteria:

- Infants aged >12 months
- Infants screening positive for NDI (using MDAT) but not meeting the criteria for moderate-severe NDI on GMDC & HINE assessment
- Conditions requiring prolonged inpatient treatment
- Parents unwilling or unable to attend the full programme
- Main residence outside Nakaseke or Luwero district, and >20km from Mulago Hospital

material to ensure internal consistency in assessments technique. Inclusion and exclusion criteria are outlined in Box 1.

Baseline characteristics

Infant and caregiver demographic information will be recorded at baseline, including date of birth, age, sex, birth order, parity, antepartum, intrapartum and postpartum history, family and medical history, developmental history, mother's education and occupation, family details including family size, and ages, household incomes, household SES and residence. All outcome measures will also be measured at baseline enabling pre- and post-intervention comparisons.

Randomisation

Infants and their caregivers will be randomised in a 1:1 ratio to either the EIP or SC arm. Randomisation will be stratified by recruitment centre. Randomisation lists indicating a randomisation number and trial arm allocation, will be prepared by the trial statistician using a random number generator in Stata (version 15) prior to the commencement of the study, and stored on a secure, password-protected computer at the Medical Research Council/Uganda Virus Research Institute & LSHTM Uganda Research Unit (MRC/UVRI) by a statistician otherwise not involved in the study. When a participant is eligible for recruitment and consent obtained, study staff will contact the MRC/UVRI statistician who

For peer review only - http://bmjopen.bmj.com/site/about/guidelines.xhtml

will inform the study staff of the study number and trial arm to which the participant is to be allocated. The personnel in charge of the randomisation will not be involved in other study procedures, including assessment of outcomes.

Intervention arm

Box 2. Developing the ABAaNA early intervention programme

In LMICs, services for affected children are often lacking and parental levels of knowledge and understanding about cerebral palsy are often low. To fill this gap, a parent training programme called 'Getting to Know Cerebral Palsy' was developed and launched in partnership between the LSHTM and CBM (Christian Blind Mission) an international disability and development organisation (https://www.ubuntu-hub.org). The programme aims to increase parental knowledge and skills and promotes a participatory learning approach with an emphasis on the empowerment of caregivers across a broad spectrum of impairment for children aged 2-12 years.(17, 18)

Since 2011, the ABAaNA studies ('Abaana', meaning 'children in the local language Luganda) have been examining risk factors for, and outcomes from, neonatal encephalopathy (NE) in Uganda.(15) Studies examining early neurodevelopmental outcomes after NE revealed a high prevalence of NDI with 25% of those affected also having malnutrition from related feeding difficulties.(15) Qualitative work highlighted the stigma and broad-ranging social, emotional and financial impacts on affected families,(3) and the need for an intervention that may improve life chances amongst affected families.

The EIP was developed around the principles of 'Getting to Know Cerebral Palsy' (http://ubuntu-hub.org), and has been adapted for younger children aged 0-2 years following an iterative process following MRC recommendations on development and evaluation of complex interventions,(19); it was supported by a diverse Expert Advisory Group including local parents with children with NDI, Disabled Persons Organisations and experts in early intervention and child development. Core themes running through the programme are summarised in Figure 2. The newly developed programme was piloted amongst 28 families at Mulago Hospital in Kampala in 2015-6 and showed a 25% improvement in family quality of life scores (PedsQL, Family Impact module 2.0) post intervention (verbal communication).

The early intervention programme is a community-based, peer-led group programme with caregivers at a community level, using a participatory approach driven by adult learning theory.(16) The programme manual is freely available to download (https://www.ubuntu-hub.org). Development of the programme is described in Box 2.

For peer review only - http://bmjopen.bmj.com/site/about/guidelines.xhtml

Participating families are encouraged to share experiences through discussion and reflection, prioritise problems and identify solutions together. Facilitators of the group sessions are ‘expert parents’, themselves parents of children with NDI, who have undergone five days of core training followed by regular supervision, face-to-face mentoring meetings and telephone discussions with existing in-country Master Facilitators (trained therapists in Uganda). Each EIP group involves 6-10 families; groups are selected depending on locality for ease of attendance. The training is divided into ten modules covering understanding disability, positioning and carrying, feeding, mobilising, communication, play, everyday activities, and experiences in the local community (Figure 2, Table 1). Individual module sessions are delivered every 1-2 weeks and last 2-3 hours including time for facilitated discussion; the entire programme is designed to be delivered over six months including at least one home visit conducted by the expert parent facilitator.

Figure 2: Core themes and content of the ABAaNA Early Intervention Programme

Table 1: Description of the programme modules

Module	Content
1. Let's get started	Content and ground rules of the programme<br>Understanding cerebral palsy, additional resources for information<br>Personal stories
2. Know your child	Developmental milestones for young children<br>Determining each child's progress
3. Positioning and carrying	The importance of optimal positioning<br>Practical skills regarding optimal positioning
4. Eating and drinking	Feeding challenges for children with neurodevelopmental impairment<br>Practical skills for addressing feeding challenges
5. Learning to move	Understanding different types of movement<br>Practical skills for assisting learning to move
6. Communicating	The importance of communication<br>Practical advice to encourage their child to communicate
7. Play and early stimulation	The importance of early stimulation and play for children to develop<br>Challenges of inclusion in play with the family and community<br>Creation of simple toys<br>How parents/ caregivers can encourage their child to play
8. Everyday activities	Using everyday activities to promote child development<br>Management of seizures<br>Review of previous sessions
9. Our community	Community resources available<br>Discussion around barriers to inclusion, addressing stigma and discrimination<br>Understanding disability rights<br>Thoughts and feelings of the caregiver<br>Members of community invited to attend this session
10. Next steps	Planning to facilitate their own group<br>Reflection on learning points

For peer review only - http://bmjopen.bmj.com/site/about/guidelines.xhtml

	Endpoint data collection

Fidelity & Adherence to the intervention

EIP facilitators will receive a 5-day training programme delivered by two Master Facilitators, which includes facilitation skills, knowledge transfer on the core contents of the EIP manual and translation of knowledge to practice through simulated sessions with families and children with NDI. All trial intervention groups will be co-facilitated by a Master Facilitator providing supportive supervision to new facilitators. After each modular group meeting, a short-facilitated feedback session will be conducted, and the content of the module delivered will be recorded. Attendance of individual caregivers and children at the group sessions will be recorded. Facilitators will emphasize to caregivers the importance of attending all sessions, with phone calls prior to each session to promote adherence. If missed, a catch-up session may be offered before the next module.

Standard care arm

SC refers to care that is currently available in established local services. In both sites this includes referral to physiotherapy, seizure management and nutritional support. Information on access to local medical, therapy and family services will be collected. Families in the SC arm will be offered delayed entry into the EIP after completing the 18-month assessment. Contamination of the SC arm by exposure of SC families to intervention will be monitored and reported.

Outcomes

Participants in both arms will be assessed by study staff masked to trial allocation at two time points; at age 12-17 months (which corresponds to completion of the EIP in the intervention arm, 6 months after recruitment) and again at age 18-23 months (12 months after recruitment). (Figure 1). Caregivers will be phoned a week before the follow-up assessments to arrange a time for interview. Assessments will be primarily conducted in the study-site clinics. Where caregivers cannot be contacted by phone or are unable to attend the clinic, a community visit will be arranged, and assessments completed at home. Outcome assessments will be conducted by Mulago assessors for children recruited at Kiwoko, and vice versa to ensure assessors are blind to allocation arm. Two assessors will independently assess a small proportion of the children and Inter-rater reliability will be calculated.

Primary outcome measures

The primary outcomes of the study will be:

For peer review only - http://bmjopen.bmj.com/site/about/guidelines.xhtml

1. *Feasibility of participant recruitment and randomisation* as assessed by the total number recruited and randomised to each arm. Qualitative tools will also be used to capture information on feasibility.
2. *Acceptability of the EIP amongst caregivers and health care workers* as assessed by the protocol violation rate (e.g. participants in the intervention arm being treated as if they were in the control arm or vice versa) at programme completion, and by the number of programme sessions attended between baseline and programme completion. Qualitative tools will also be used to capture information on acceptability.
3. *Preliminary evidence of impact on Family Quality of Life* as assessed using the scored Pediatric Quality of Life Family Impact module (PedsQL),(20) The PedsQL comprises 36 items scored on a 0-4 Likert scale and linearly transformed to a 0-100 scale, with higher scores indicating a better QoL. It will be translated into the local language Luganda and administered as a standardised structured interview by trained study staff.

Other outcomes of interest

1. *Child motor functioning* as assessed by the mobility score of the Pediatric Evaluation Disability Inventory (PEDI).(21) The PEDI is a standardised test designed to identify and describe functional impairment and monitor progress. Normative scaled scores are obtained for children ≥6 months to provide age-related expectations of ability.
2. *Child cognitive function* as assessed by the GMDS.(13)
3. *Child growth, health and well-being* assessed using weight, height and head circumference measured according to standardised protocol. Occipito-frontal head circumference (OFC, paper tape measure), weight, (SECA336 electronic scales, Hamburg, Germany) and height will be taken by study staff using standardised procedures. Haemoglobin will be determined on a finger prick sample using HemoCue Hb 201 (HemoCue AB, Angelholm, Sweden). A structured maternal interview in Luganda will report on caregivers concerns regarding health, growth and development of their child and episodes of illness including seizures and other neurological problems, feeding difficulties, chest infections, and treatment for malnutrition.
4. *Caregiver psychological distress* assessed using the Self-Referral Questionnaire (SRQ) and the Parenting Stress index (PSI).(22) The SRQ consists of 20 items each scored 0 (symptom absent) or 1 (symptom present) giving a total out of 20. The PSI is a 120-item inventory measuring the magnitude of caregiver stress attributable to parent-child relationship (Total Stress Scale), and situational/demographics factors outside the parent-child relationship (Life Stress Scale). These tools will be translated into Luganda.

For peer review only - http://bmjopen.bmj.com/site/about/guidelines.xhtml

5. *Caregiver-child attachment* assessed using the Maternal Infant Responsiveness Instrument (MIRI); a 22-item scale designed to measure the parent's feelings about their infant and an appraisal of the infant's responses.(23)
6. *Quality of the home environment* assessed using the Infant Toddler-Home Observation for the Measurement of the Environment (IT-HOME). This comprises 45 items, based on observation and/or interview, assessing the physical environment of the home and the child's interaction within it.(24)
7. *Cost of illness and protective effectiveness* will be assessed (separate protocol, Katumba et al)

Qualitative methods

IDIs will be conducted with five randomly selected caregivers from each arm at each site. FGDs will be conducted with caregivers, at baseline, 6 months post-recruitment and again 6 months later in both the intervention and SC arms. Amongst intervention arm families, qualitative techniques will be used to capture information on the feasibility, acceptability and impact of the EIP intervention using qualitative tools including focus group discussions (FGDs), in-depth-interviews (IDIs) and observation.

We will describe the experiences of children and caregivers relating to the intervention received including the impact of the disability, parental confidence level, inclusion in community life and experience of stigma and discrimination. We will examine changes in these domains over the follow-up period and explore attributions of change. In addition, we will perform social mapping of parent networks and group discussions with staff on their perspectives and experiences of using the EIP. The themes guiding our analysis will be drawn not only from objectives of the trial but also from the data, should additional areas of interest emerge during interviews and discussions.

The interviews will be conducted by social scientists who have experience in qualitative research.

Data management and access

Data collected in the clinic or at field visits will be entered on standard clinical record forms (CRFs). Clinical data will be recorded under a unique study ID number. Completed CRFs will be checked by and double-entered into a trial-specific MS Access database. Data from both IDIs and FGDs will be collected in the form of audiotapes, transcripts and field notes. All data entry and data management will be overseen by a statistician/data manager at the MRC/UVRI Unit. Data will be maintained on the host institution server and backed-up following standardised operating procedures. Paper CRFs will be stored in lockable filing cabinets at the sites. Access to these data during the trial will be restricted to essential personnel (the PIs, site co-investigators, medical research officers, and data clerks).

For peer review only

Confidentiality

All research-team members will receive training in confidentiality. Data will be stored without personal identifiers, except where names must be included to ensure identification of the correct participants for procedures. All data will be stored on password-protected computers, accessible only to research team members.

Sample size

The trial will recruit 126 children and their caregivers; 63 per arm. Allowing for a 20% dropout rate, this sample size will give 90% power to detect a minimal relative difference of 20% on PedQL Family Impact score between the intervention and control arms, at 5% significance level, assuming a mean PedQL score of 65 in the standard care arm and SD of 20 in both arms. Assumptions are based on data from the pilot study showing a mean caregiver PedQL score for families before the intervention of 64.9 (standard deviation (SD) 19.6) and mean score of 78.9 for families after receiving the intervention (SD 17.5).

Statistical analysis

The first primary outcome, feasibility of participant recruitment and randomisation, will be assessed by the total number recruited and randomised to each arm. Recruitment and randomisation feasibility will be demonstrated if the target sample size of 126 is achieved. Data on participants screened, eligible and randomised will be displayed in a CONSORT flowchart. Descriptive statistics (frequencies, means, medians, standard deviations and interquartile ranges) will be used to describe the sample at baseline, by trial arm

The second primary outcome, acceptability, will be assessed quantitatively by (a) calculating the protocol violation rate and (b) summarising the number of programme sessions attended between baseline and programme completion for those in the intervention arm. Protocol violation rate will be calculated as the number of participants for whom one or more protocol violations occur divided by the total number of participants, and will be presented both overall, and by trial arm. For participants in the EIP trial arm, the overall number of modules attended by each participant will be tabulated. Acceptability on the basis of number of programme sessions will be defined as attendance of at least 6 modules.

For the third primary outcome and secondary outcomes, analyses will compare outcomes between intervention and control arms at the end of the programme, when the participants will be aged 12-17 months, and again 6 months later. Analysis will be on an intention-to-treat basis and missing data

For peer review only - http://bmjopen.bmj.com/site/about/guidelines.xhtml

will not be imputed. Data for each outcome measure will be summarised by trial arm, using proportions for binary outcomes and means or medians for quantitative outcomes, depending on normality of the distribution. Differences in means/proportions between trial arms together with 95% confidence intervals will be calculated. We do not plan any formal statistical tests due to the preliminary nature of the trial; instead confidence intervals will provide a possible range of effect sizes. Regression models (linear regression for continuous outcomes, logistic regression for binary outcomes) will be used to adjust comparisons for baseline measures of the outcomes, which were collected at enrolment into the trial, in order to improve precision of effectiveness estimates. For skewed continuous outcomes, data will be normalised before analysis using suitable transformations or quantile regression will be considered. No subgroup analyses are planned.

Qualitative data will be analysed using a thematic framework approach. Themes will be based on the study objectives and those emerging from the data. Social scientists (two people) will agree the coding frame and undertake analysis collaboratively to ensure agreement on the coding approach. Thematic summaries will be developed and shared with the wider team for discussion.

Trial management, data monitoring and reporting of adverse events

The TSC (25) will oversee progress of the study towards its objectives, review relevant information from other sources (e.g. other related trials) and receive reports from the Data and Safety Monitoring Board (DSMB). All adverse events, whether related to the intervention or not, will be noted and reported. A Data Monitoring and Safety Committee (DSMC) has been established independent of the investigators and the TSC but reporting to the TSC and the sponsor. The DSMB includes an expert on global child heath, a senior statistician, and a senior academic working in newborn and early child health research in Uganda, independent of the investigators. The DSMB will have access to all data on request. Resulting from the initial meeting of the DSMB on 28th June 2017, no formal stopping rules will be applied.

Children with NDI and particularly those with seizure disorders and difficulties with swallowing, are at increased mortality risk. All adverse events, whether related to the intervention or not, will be investigated and reported according to the UVRI Research Ethics Committee in accordance with GCP requirements. All deaths, hospitalisations and other serious adverse effects will be reported to the relevant ethics committee irrespective of whether the death or event is related to disease progression or not. Trial data monitoring will be conducted by an internal independent monitor at initiation, 6 months into data collection, again after one year and end of data collection.

For peer review only - http://bmjopen.bmj.com/site/about/guidelines.xhtml

For peer review only

Participant and public involvement

The intervention, study design and conduct, were developed directly from the engagement of caregivers and programme facilitators ('expert parents') with a parent representative on the TSC. The priorities and experiences of caregivers identified during facilitated group discussions at a key-stakeholders meeting (June 2017) contributed to the development of our research question and outcome measures. Plans to communicate findings to participants and the wider community will involve caregivers, through formal discussions with the TSC.

Ethics & Dissemination

Ethics

The protocol has been approved by the Research and Ethics committee of the UVRI, Mulago Hospital and Kiwoko Hospitals, the Uganda National Council for Science and Technology, the Uganda President's Office, and the ethics committee of the LSHTM. Information sheets will be available in English and Luganda, the main local language. Parents will be provided with an oral and written explanation of the study by Ugandan study staff to ensure that information is accessible to those with lower levels of literacy. Witnessed consent using a thumb print will be available to parents/guardians who are non-literate. Reimbursement for the cost of transport will be provided to caregivers on attendance at the screening and recruitment visits.

All recruited children will receive SC at the study sites. This will include referral to local services for seizure management and physiotherapy where available. To date the benefits of the proposed EIP have not been proven and may have a negative effect if children are incorrectly classified as having NDI and placed in the programme. Children and their caregivers in the control arm will receive delayed entry into the programme for older children ('Getting to Know Cerebral Palsy') at 18-23 months at the time of their final study assessments.

Dissemination

Our programme has strong links with partnership organisations working in Maternal and Child Health programming including Adara Development, Kiwoko Hospital, Nakaseke District Health Office and other collaborating institutions. Research findings will be disseminated to the Ministry of Health, to inform local and national health policies. Regional level stakeholders including the Nakaseke District Health Office and heads of regional health and social services, will be engaged to support staff recruitment, contributing to the sustainability of the innovation at local and district level. Meetings for key-stakeholders, including local NGOs working in child disability will be held twice

For peer review only - http://bmjopen.bmj.com/site/about/guidelines.xhtml

during the project period to promote buy-in, facilitate fast-cycle learning, disseminate study findings and ultimately promote sustainability of the programme. Global learning will be facilitated through our existing online community-of-practice spanning 70 countries and >300 members. Communications support staff at MRC/UVRI, LSHTM and Adara Development will facilitate dissemination of information through appropriate media outlets, the web and social media.

Study findings will be published through Open Access peer-reviewed journals, presentations at local, national and international conferences and to the local community through community meetings. Written reports will be submitted to UVRI REC and reported to the trial registry. Data will be made available upon request.

Authors contributions

The study was conceived and designed by CT with substantial contribution from MN, DL, JN, EW, CM, JS, KK and FC. Research methodology was developed by MN, CN, MZ, JN, BM, DK, SS, RN, MS, FC, MMB, AM and CT. The first version of the paper was written by CT, SS & MN. All authors contributed to the final version of the manuscript.

Acknowledgements

The authors would like to thank the members of the ABAaNA Early Intervention Programme Expert Advisory Committee who supported the development of the programme and the implementation partners Kiwoko Hospital, Adara Development and Mulago University Hospital. They would like to acknowledge the contribution of Ellipsis for formatting the programme manual and thank Joanna Lawn for her contribution to manual illustrations. The authors would like to thank the funders, Grand Challenges Canada, Saving Brains and the Research Foundation of Cerebral Palsy Alliance Australia.

Funding statement

This work is supported by Saving Brains, Grand Challenges Canada grant number 1707-08687. The economic evaluation is supported by a Project Grant, awarded by the Research Foundation, Cerebral Palsy Alliance (PG02917).

The Trial sponsor is the MRC/UVRI and LSHTM Uganda Research Unit, Entebbe; Contact name: Prof Pontiano Kaleebu (Director); Address: MRC/UVRI & LSHTM Uganda Research Unit, Plot 51-59 Nakiwogo Road, P.O. Box 49 Entebbe, UGANDA Tel: +256 (0) 417 704000; mrc@mrcuganda.org

For peer review only - http://bmjopen.bmj.com/site/about/guidelines.xhtml

The funder had no role in the research design and will not have any role in the execution, analyses, interpretation of the data, or decision to submit results.

Data Sharing Statement

This is a study protocol and therefore no unpublished data are yet available. Data collected during the course of the trial will be made available on request, after the main study findings have been accepted for publication. Competing interest statement

We declare no competing interests.

References

1. WHO U. Survive and Thrive: Transforming Care for Every Small and Sick Newborn. Key Findings 2018 [Available from: https://www.unicef.org/every-child-alive/Survive-and-Thrive_KEY_FINDINGS_FINAL.pdf.
2. Olusanya BO, Davis AC, Wertlieb D, Boo N-Y, Nair MKC, Halpern R, et al. Developmental disabilities among children younger than 5 years in 195 countries and territories, 1990–2016: a systematic analysis for the Global Burden of Disease Study 2016. The Lancet Global Health. 2018;6(10):e1100-e21.
3. Nakamanya S, Siu GE, Lassman R, Seeley J, Tann CJ. Maternal experiences of caring for an infant with neurological impairment after neonatal encephalopathy in Uganda: a qualitative study. Disability and rehabilitation. 2015;37(16):1470-6.
4. Wallander JL, McClure E, Biasini F, Goudar SS, Pasha O, Chomba E, et al. Brain research to ameliorate impaired neurodevelopment--home-based intervention trial (BRAIN-HIT). BMC Pediatr. 2010;10:27.
5. Morgan C, Darrah J, Gordon AM, Harbourne R, Spittle A, Johnson R, et al. Effectiveness of motor interventions in infants with cerebral palsy: a systematic review. Developmental medicine and child neurology. 2016;58(9):900-9.
6. Palmer FB, Shapiro BK, Wachtel RC, Allen MC, Hiller JE, Harryman SE, et al. The effects of physical therapy on cerebral palsy. A controlled trial in infants with spastic diplegia. N Engl J Med. 1988;318(13):803-8.
7. Bao X, Sun S, Wei S. Early intervention promotes intellectual development of premature infants: a preliminary report. Early Intervention of Premature Infants Cooperative Research Group. Chin Med J (Engl). 1999;112(6):520-3.
8. Gardner JM, Walker SP, Powell CA, Grantham-McGregor S. A randomized controlled trial of a home-visiting intervention on cognition and behavior in term low birth weight infants. J Pediatr. 2003;143(5):634-9.
9. Walker SP, Chang SM, Powell CA, Grantham-McGregor SM. Psychosocial intervention improves the development of term low-birth-weight infants. J Nutr. 2004;134(6):1417-23.
10. Carlo WA, Goudar SS, Pasha O, Chomba E, Wallander JL, Biasini FJ, et al. Randomized trial of early developmental intervention on outcomes in children after birth asphyxia in developing countries. J Pediatr. 2013;162(4):705-12 e3.
11. Benfer KA, Novak I, Morgan C, Whittingham K, Khan NZ, Ware RS, et al. Community-based parent-delivered early detection and intervention programme for infants at high risk of cerebral palsy in a low-resource country (Learning through Everyday Activities with Parents (LEAP-CP): protocol for a randomised controlled trial. BMJ open. 2018;8(6):e021186.

For peer review only

12. Gladstone M, Lancaster GA, Umar E, Nyirenda M, Kayira E, van den Broek NR, et al. The Malawi Developmental Assessment Tool (MDAT): the creation, validation, and reliability of a tool to assess child development in rural African settings. PLoS Med. 2010;7(5):e1000273.
13. Griffiths R, Huntley M. The Griffiths mental development scales : from birth to two years : manual. Henley on Thames: ARICD; 1996.
14. Haataja L, Mercuri E, Regev R, Cowan F, Rutherford M, Dubowitz V, et al. Optimality score for the neurologic examination of the infant at 12 and 18 months of age. J Pediatr. 1999;135(2 Pt 1):153-61.
15. Tann CJ, Webb EL, Lassman R, Ssekyewa J, Sewegaba M, Musoke M, et al. Early Childhood Outcomes After Neonatal Encephalopathy in Uganda: A Cohort Study. EClinicalMedicine. 2018;6:26-35.
16. Knowles M. The Adult Learning Theory - Andragogy 1984 [Available from: https://elearningindustry.com/the-adult-learning-theory-andragogy-of-malcolm-knowles.
17. Zuurmond M, O'Banion D, Gladstone M, Carsamar S, Kerac M, Baltussen M, et al. Evaluating the impact of a community-based parent training programme for children with cerebral palsy in Ghana. PloS one. 2018;13(9):e0202096.
18. Zuurmond M, Nyante G, Baltussen M, Seeley J, Abanga J, Shakespeare T, et al. A support programme for caregivers of children with disabilities in Ghana: Understanding the impact on the wellbeing of caregivers. Child Care Health Dev. 2019;45(1):45-53.
19. Campbell M, Fitzpatrick R, Haines A, Kinmonth AL, Sandercock P, Spiegelhalter D, et al. Framework for design and evaluation of complex interventions to improve health. BMJ. 2000;321(7262):694-6.
20. Varni JW, Burwinkle TM, Seid M, Skarr D. The PedsQL 4.0 as a pediatric population health measure: feasibility, reliability, and validity. Ambul Pediatr. 2003;3(6):329-41.
21. Haley SM, Coster WJ, Ludlow LH, Haltiwanger JT, Andrellos PJ. Pediatric Evaluation of Disability Inventory. Pediatric Physical Therapy Pediatric Physical Therapy. 1994;6(1):42.
22. Abidin RR. Parenting Stress Index. Lutz, FL: PAR; 2012.
23. Amankwaa L. The Maternal Infant Responsiveness Instrument 2002 [Available from: https://miriinstrument.wordpress.com/.
24. Caldwell BM, Bradley RH, University of Arkansas at Little R, Center for Child D, Education. Administration manual : HOME observation for measurement of the environment. Little Rock, Ark.: University of Arkansas at Little Rock; 1984.
25. MRC. Guidelines for good clinical practice in clinical trials 1998 [Available from: https://mrc.ukri.org/documents/pdf/good-clinical-practice-in-clinical-trials/.

For peer review only

For peer review only - http://bmjopen.bmj.com/site/about/guidelines.xhtml

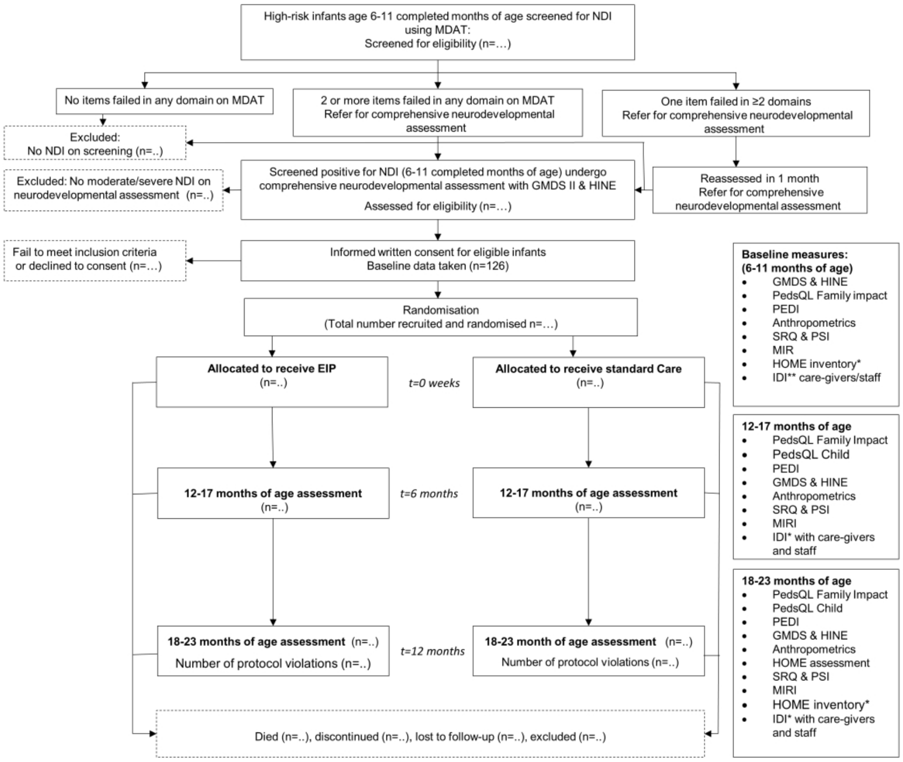

*In-depth interviews (IDI) with caregivers on impact of disability, confidence level of the parents, level of participation in family and community life and experience of stigma/discrimination. MDAT=Malawi Developmental Assessment Tool, PedQL=Pediatric Quality of Life tool, PEDI=Pediatric Evaluation Disability Inventory GMDS=Griffiths Mental Developmental Scales, HINE=Hammersmith Infant Neurological Examination, HOME=Home Observation for the Measurement of the Environment, SRQ=Self-Referral Questionnaire, PSI=Parent Stress Index MIRI=Maternal Infant Responsiveness Inventory

309x339mm (300 x 300 DPI)

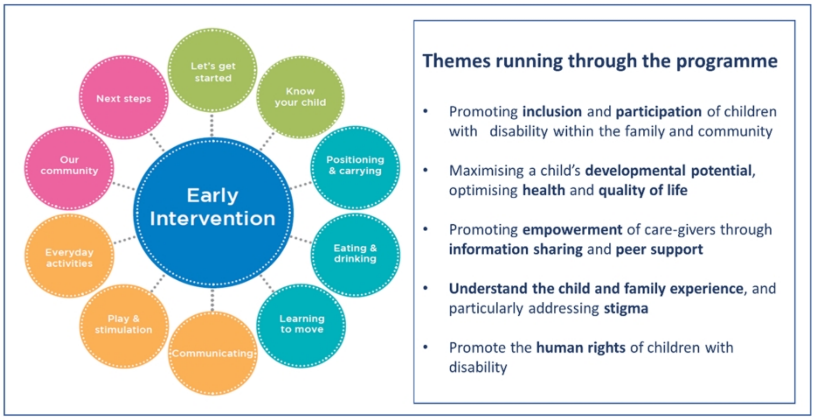

Figure 2: Core themes and content of the ABAaNA Early Intervention Programme

338x190mm (300 x 300 DPI)

STANDARD PROTOCOL ITEMS: RECOMMENDATIONS FOR INTERVENTIONAL TRIALS

SPIRIT 2013 Checklist: Recommended items to address in a clinical trial protocol and related documents*

Section/item	Item No	Description	Addressed on page number
**Administrative information**			
Title	1	Descriptive title identifying the study design, population, interventions, and, if applicable, trial acronym	_______1________
Trial registration	2a	Trial identifier and registry name. If not yet registered, name of intended registry	_______2________
	2b	All items from the World Health Organization Trial Registration Data Set	_______N/A_____
Protocol version	3	Date and version identifier	_______2_______
Funding	4	Sources and types of financial, material, and other support	_______16____
Roles and responsibilities	5a	Names, affiliations, and roles of protocol contributors	_____1 &_______
	5b	Name and contact information for the trial sponsor	______1________
	5c	Role of study sponsor and funders, if any, in study design; collection, management, analysis, and interpretation of data; writing of the report; and the decision to submit the report for publication, including whether they will have ultimate authority over any of these activities	_______16____
	5d	Composition, roles, and responsibilities of the coordinating centre, steering committee, endpoint adjudication committee, data management team, and other individuals or groups overseeing the trial, if applicable (see Item 21a for data monitoring committee)	_____13/14____

For peer review only - http://bmjopen.bmj.com/site/about/guidelines.xhtml

Introduction

Background and rationale	6a	Description of research question and justification for undertaking the trial, including summary of relevant studies (published and unpublished) examining benefits and harms for each intervention	______4______
	6b	Explanation for choice of comparators	______4___
Objectives	7	Specific objectives or hypotheses	______5______
Trial design	8	Description of trial design including type of trial (eg, parallel group, crossover, factorial, single group), allocation ratio, and framework (eg, superiority, equivalence, noninferiority, exploratory)	_____4-5______

Methods: Participants, interventions, and outcomes

Study setting	9	Description of study settings (eg, community clinic, academic hospital) and list of countries where data will be collected. Reference to where list of study sites can be obtained	_____5-6_____
Eligibility criteria	10	Inclusion and exclusion criteria for participants. If applicable, eligibility criteria for study centres and individuals who will perform the interventions (eg, surgeons, psychotherapists)	_____7 (Box 1)_
Interventions	11a	Interventions for each group with sufficient detail to allow replication, including how and when they will be administered	____8-10_____
	11b	Criteria for discontinuing or modifying allocated interventions for a given trial participant (eg, drug dose change in response to harms, participant request, or improving/worsening disease)	____N/A_____
	11c	Strategies to improve adherence to intervention protocols, and any procedures for monitoring adherence (eg, drug tablet return, laboratory tests)	____10_______
	11d	Relevant concomitant care and interventions that are permitted or prohibited during the trial	____10_______
Outcomes	12	Primary, secondary, and other outcomes, including the specific measurement variable (eg, systolic blood pressure), analysis metric (eg, change from baseline, final value, time to event), method of aggregation (eg, median, proportion), and time point for each outcome. Explanation of the clinical relevance of chosen efficacy and harm outcomes is strongly recommended	____10-11____
Participant timeline	13	Time schedule of enrolment, interventions (including any run-ins and washouts), assessments, and visits for participants. A schematic diagram is highly recommended (see Figure)	__10 &Figure 2

For peer review only - http://bmjopen.bmj.com/site/about/guidelines.xhtml

Sample size	14	Estimated number of participants needed to achieve study objectives and how it was determined, including clinical and statistical assumptions supporting any sample size calculations	___12-13_____
Recruitment	15	Strategies for achieving adequate participant enrolment to reach target sample size	___6-7______
**Methods: Assignment of interventions (for controlled trials)**			
Allocation:			
Sequence generation	16a	Method of generating the allocation sequence (eg, computer-generated random numbers), and list of any factors for stratification. To reduce predictability of a random sequence, details of any planned restriction (eg, blocking) should be provided in a separate document that is unavailable to those who enrol participants or assign interventions	____7-8______
Allocation concealment mechanism	16b	Mechanism of implementing the allocation sequence (eg, central telephone; sequentially numbered, opaque, sealed envelopes), describing any steps to conceal the sequence until interventions are assigned	____7-8_____
Implementation	16c	Who will generate the allocation sequence, who will enrol participants, and who will assign participants to interventions	_____7-8______
Blinding (masking)	17a	Who will be blinded after assignment to interventions (eg, trial participants, care providers, outcome assessors, data analysts), and how	___2,5,10______
	17b	If blinded, circumstances under which unblinding is permissible, and procedure for revealing a participant's allocated intervention during the trial	____13-14______
**Methods: Data collection, management, and analysis**			
Data collection methods	18a	Plans for assessment and collection of outcome, baseline, and other trial data, including any related processes to promote data quality (eg, duplicate measurements, training of assessors) and a description of study instruments (eg, questionnaires, laboratory tests) along with their reliability and validity, if known. Reference to where data collection forms can be found, if not in the protocol	____6-11______
	18b	Plans to promote participant retention and complete follow-up, including list of any outcome data to be collected for participants who discontinue or deviate from intervention protocols	____10______

Data management	19	Plans for data entry, coding, security, and storage, including any related processes to promote data quality (eg, double data entry; range checks for data values). Reference to where details of data management procedures can be found, if not in the protocol	_____12______
Statistical methods	20a	Statistical methods for analysing primary and secondary outcomes. Reference to where other details of the statistical analysis plan can be found, if not in the protocol	_____13_______
	20b	Methods for any additional analyses (eg, subgroup and adjusted analyses)	_______N/A_____
	20c	Definition of analysis population relating to protocol non-adherence (eg, as randomised analysis), and any statistical methods to handle missing data (eg, multiple imputation)	_______13_______
**Methods: Monitoring**			
Data monitoring	21a	Composition of data monitoring committee (DMC); summary of its role and reporting structure; statement of whether it is independent from the sponsor and competing interests; and reference to where further details about its charter can be found, if not in the protocol. Alternatively, an explanation of why a DMC is not needed	_____13-14____
	21b	Description of any interim analyses and stopping guidelines, including who will have access to these interim results and make the final decision to terminate the trial	____13-14_____
Harms	22	Plans for collecting, assessing, reporting, and managing solicited and spontaneously reported adverse events and other unintended effects of trial interventions or trial conduct	_____14______
Auditing	23	Frequency and procedures for auditing trial conduct, if any, and whether the process will be independent from investigators and the sponsor	____14_______
**Ethics and dissemination**			
Research ethics approval	24	Plans for seeking research ethics committee/institutional review board (REC/IRB) approval	____14_______
Protocol amendments	25	Plans for communicating important protocol modifications (eg, changes to eligibility criteria, outcomes, analyses) to relevant parties (eg, investigators, REC/IRBs, trial participants, trial registries, journals, regulators)	_____15______

For peer review only - http://bmjopen.bmj.com/site/about/guidelines.xhtml

Consent or assent	26a	Who will obtain informed consent or assent from potential trial participants or authorised surrogates, and how (see Item 32)	_____14______
	26b	Additional consent provisions for collection and use of participant data and biological specimens in ancillary studies, if applicable	_____N/A________
Confidentiality	27	How personal information about potential and enrolled participants will be collected, shared, and maintained in order to protect confidentiality before, during, and after the trial	_____12________
Declaration of interests	28	Financial and other competing interests for principal investigators for the overall trial and each study site	_____16________
Access to data	29	Statement of who will have access to the final trial dataset, and disclosure of contractual agreements that limit such access for investigators	_____15________
Ancillary and post-trial care	30	Provisions, if any, for ancillary and post-trial care, and for compensation to those who suffer harm from trial participation	______14-15____
Dissemination policy	31a	Plans for investigators and sponsor to communicate trial results to participants, healthcare professionals, the public, and other relevant groups (eg, via publication, reporting in results databases, or other data sharing arrangements), including any publication restrictions	_____15________
	31b	Authorship eligibility guidelines and any intended use of professional writers	_____16________
	31c	Plans, if any, for granting public access to the full protocol, participant-level dataset, and statistical code	____15_________
**Appendices**			
Informed consent materials	32	Model consent form and other related documentation given to participants and authorised surrogates	__online______
Biological specimens	33	Plans for collection, laboratory evaluation, and storage of biological specimens for genetic or molecular analysis in the current trial and for future use in ancillary studies, if applicable	_____N/A________

*It is strongly recommended that this checklist be read in conjunction with the SPIRIT 2013 Explanation & Elaboration for important clarification on the items. Amendments to the protocol should be tracked and dated. The SPIRIT checklist is copyrighted by the SPIRIT Group under the Creative Commons "Attribution-NonCommercial-NoDerivs 3.0 Unported" license.

For peer review only - http://bmjopen.bmj.com/site/about/guidelines.xhtml

BMJ Open

A Randomised Controlled Pilot Feasibility Trial of an Early Intervention Programme for Young Infants with Neurodevelopmental Impairment in Uganda: Study Protocol

Journal:	*BMJ Open*
Manuscript ID	bmjopen-2019-032705.R2
Article Type:	Protocol
Date Submitted by the Author:	31-Aug-2019
Complete List of Authors:	Nampijja, Margaret; MRC/UVRI Uganda Research Unit On AIDS; London School of Hygiene and Tropical Medicine<br>Webb, Emily; London School of Hygiene and Tropical Medicine Faculty of Epidemiology and Population Health<br>Nanyunja, Carol; MRC/UVRI Uganda Research Unit On AIDS<br>Sadoo, Samantha; London School of Hygiene and Tropical Medicine Faculty of Epidemiology and Population Health<br>Nalugya, Ruth; Medical Research Council / Uganda Virus Research Institute & London School of Hygiene & Tropical Medicine Uganda Research Unit<br>Nyonyintono, James; Kiwoko Hospital; Adara Development<br>Muhumuza, Anita; Mulago National Referral Hospital<br>Ssekidde, Moses; Kiwoko Hospital<br>Katumba, Kenneth; MRC/UVRI Uganda Research Unit On AIDS<br>Magnusson, Brooke; Adara Development, 300 Admiral Way, # 106, Edmonds<br>Kabugo, Daniel; Adara Development<br>Cowan, Frances; Imperial College London, Depart of Paediatrics<br>Martinez-Biarge, Miriam; Imperial College London, Depart of Paediatrics<br>Zuurmond, Maria; London School of Hygiene and Tropical Medicine Faculty of Infectious and Tropical Diseases<br>Morgan, Cathy; Cerebral Palsy Alliance<br>Lester, Deborah; Seattle Children's Hospital; Adara Development<br>Seeley, Janet; MRC/UVRI Uganda Research Unit On AIDS; London School of Hygiene and Tropical Medicine Faculty of Public Health and Policy<br>Tann, Cally; London School of Hygiene and Tropical Medicine Faculty of Epidemiology and Population Health; MRC/UVRI Uganda Research Unit On AIDS
**Primary Subject Heading**:	Paediatrics
Secondary Subject Heading:	Global health, Qualitative research, Rehabilitation medicine
Keywords:	Neonatal encephalopathy, Uganda, Neurodevelopmental impairment, Cohort study, Child disability, Developmental disability

SCHOLARONE™
Manuscripts

A Randomised Controlled Pilot Feasibility Trial of an Early Intervention Programme for Young Infants with Neurodevelopmental Impairment in Uganda: Study Protocol

Authors: Margaret Nampijja[1], Emily L Webb[2], Carol Nanyunja[1], Samantha Sadoo[3], Ruth Nalugya[1], James Nyonyintono[4,5], Anita Muhumuza[6], Moses Ssekidde[4], Kenneth R. Katumba[1], Brooke Magnusson[5], Daniel Kabugo[5], Frances M Cowan[7], Miriam Martinez-Biarge[7], Maria Zuurmond[8], Cathy Morgan[9,10], Debbie Lester [5,11], Janet Seeley[1,12], Cally Tann [1,3,13*]

Affiliations

[1]MRC/UVRI & LSHTM Uganda Research Unit, Plot 51-59 Nakiwogo Road, Entebbe, Uganda (Trial Sponsor)

[2]MRC Tropical Epidemiology Group, London School of Hygiene and Tropical Medicine, Keppel Street, London, UK

[3]Department of Infectious Disease Epidemiology, School of Hygiene and Tropical Medicine, Keppel Street, London, UK

[4]Kiwoko Hospital, PO Box 149, Nakaseke, Uganda

[5]Adara Development, 300 Admiral Way, #106, Edmonds, Washington, USA

[6]Mulago National Referral Hospital, Makerere Hill Road, Kampala, Uganda

[7]Department of Paediatrics, Imperial College London, London, UK

[8]Department of Infectious & Tropical Diseases, London School of Hygiene and Tropical Medicine, Keppel Street, London, UK

[9]Paediatrics and Child Health, School of Medicine, University of Sydney, Sydney Australia

[10]Cerebral Palsy Alliance Research Institute, Sydney, Australia

[11]Seattle Children's Hospital, 4800 Sand Point Way NE, Seattle, Washington, USA

[12]Department of Global Health & Development, London School of Hygiene & Tropical Medicine, 15-17 Tavistock Place, London, UK

[13]Neonatal Medicine, University College London Hospitals NHS Trust, 235 Euston Rd, London, UK

*Corresponding author:

Dr Cally Tann

Email: cally.tann@lshtm.ac.uk

Tel: +44 (0) 7967 367 004

MARCH Centre, London School of Hygiene and Tropical Medicine, Keppel Street, London. WC1E 7HT

Key words: Neonatal encephalopathy, Uganda, outcomes, impairment, neurodevelopment, cohort study

For peer review only - http://bmjopen.bmj.com/site/about/guidelines.xhtml

ABSTRACT

Introduction:

Early intervention programmes for infants with neurodevelopmental impairment have been poorly studied especially in low-income settings. We aim to evaluate the feasibility and acceptability of a group participatory early intervention programme, the 'ABAaNA Early Intervention Programme' (EIP), for young children with neurodevelopmental impairment in Uganda.

Methods & Analysis:

We will conduct a pilot feasibility, single-blinded, randomised controlled trial comparing the EIP with standard care across two study sites (one urban, one rural) in central Uganda. Eligible infants (n=126, age 6-11 completed months) with neurodevelopmental impairment (defined as a developmental quotient <70 on Griffiths Scales of Mental Development, and, or Hammersmith Infant Neurological Examination score <60 will be recruited and randomised to the intervention or standard care arm. Intervention arm families will receive the 10-modular, peer-facilitated, participatory, community-based programme over 6 months. Recruited families will be followed up at 6 and 12 months after recruitment, and assessors will be blinded to the trial allocation. The primary hypothesis is that the ABAaNA EIP is feasible and acceptable when compared to standard care. Primary outcomes of interest are feasibility (number recruited and randomised at baseline) and acceptability (protocol violation of arm allocation and number of sessions attended) and family and child quality of life. Guided by the study aim, the qualitative data analysis will use a data-led thematic framework approach. The findings will inform scalability and sustainability of the programme.

Ethics & Dissemination: The trial protocol has been approved by the relevant Ugandan and UK ethics committees. Recruited families will give written informed consent and we will follow international codes for ethics and good clinical practice. Dissemination will be through peer-reviewed publications, conference presentations, and public engagement.

Trial Registration: ISRCTN44380971; protocol version 3.0, 19th February 2018

For peer review only - http://bmjopen.bmj.com/site/about/guidelines.xhtml

Article Summary

Strengths and limitations of this study

- This pilot feasibility trial is amongst the first to examine feasibility and acceptability of an early intervention programme for young children with neurodevelopmental impairment in a low resource sub-Saharan African setting.
- The mixed-methods evaluation of this complex community-level intervention will provide important information on implementation of an early intervention programme for child disability at scale.
- Whilst the small sample size and individually randomised trial design will limit our understanding of programme impact, quantitative and qualitative data will inform design and execution of a larger future trial to examine effects on important child and family outcomes.

For peer review only

For peer review only - http://bmjopen.bmj.com/site/about/guidelines.xhtml

INTRODUCTION

Globally each year, an estimated 30 million neonates experience complications around the time of birth which can have a life-long impact on health and development.(1) The United Nations Global Strategy for Women's, Children's and Adolescents' Health (2016-2030) advocates the need for all children not only to 'survive' but also to 'thrive'.(1) Whilst in recent decades substantial progress has been made in reducing child mortality in low- and middle-income countries (LMICs), the global burden of developmental disabilities remains unchanged.(2) Child neurodevelopmental impairment (NDI) significantly impacts families in any context, but particularly in low-resource settings, where availability and access to support services are limited, financial barriers greater, and social stigma more overt.(3)

A wide spectrum of impairment is seen after newborn illnesses, including cerebral palsy, ineffective feeding, learning, visual and hearing difficulties and epilepsy.(4) A growing evidence base, largely from high-income countries (HICs), suggests that early intervention programmes (EIPs) commencing in the first months after birth, have the potential to limit and even prevent developmental and cognitive impairments following early brain injury. These programmes target the neuroplasticity of the immature developing brain, either directly or indirectly, through family capacity building and enrichment of the care-giving environment.(5)

In HICs, it has been shown that early environmental enrichment can enhance motor function in children <2 years.(4, 6) In LMICs, several trials have shown positive effects of EIPs in at-risk infants,(7-10) although these studies have not focused on infants specifically with NDI. Few studies have examined the feasibility and acceptability on affected children and their caregivers, and how they might be integrated into existing community health programmes.(11) Scalability and sustainability of an intervention programme is also dependent on its cost-effectiveness. This is particularly true in low- and middle-income countries where resources are scarce and existing care structures for children much less well established.

Aims

The study aims to evaluate whether a facilitated, community-based, participatory early intervention programme is feasible and acceptable. We will conduct a pilot feasibility single-blind, randomised-controlled trial (RCT) with two parallel groups. The outcomes of interest are feasibility of randomisation and recruitment, acceptability among caregivers and health care workers and early evidence of family impact quality of life, 6 months after recruitment and again 6 months later. The incremental and protective cost-effectiveness of the EIP and the economic impact of child

For peer review only

developmental disability to families and services in Uganda will be examined by Katumba et al in a separate protocol.

Objectives & Hypotheses

The primary objectives of the study are to:

1. Describe the feasibility and acceptability of the EIP for children with NDI and their families.
Hypothesis*: It will be feasible to conduct an RCT of the EIP versus standard care (SC) in rural and urban contexts and acceptable to families and the community.*

2. Obtain preliminary data on whether the EIP improves family quality of life when compared with SC.
Hypothesis: *Families receiving the community based EIP will demonstrate improved QoL scores on the Paediatric Quality of Life Family Impact module compared to SC 12 months after recruitment.*

3. Identify the main barriers and facilitating factors for scaling up the EIP.
Hypothesis*: The EIP is scalable in this low-resource Ugandan setting.*

4. Determine the incremental and protective cost-effectiveness of the ABAaNA EIP.
Hypothesis: *The ABAaNA EIP is a cost-effective intervention to improve family QoL for children with NDI.*

METHODS

We will conduct a pilot feasibility, single-blind, RCT with two parallel groups; one receiving the EIP and the other SC.

Study Setting

The study is based at two Ugandan sites; one urban (Mulago Hospital, Kampala) and one rural (Kiwoko Hospital, Nakaseke). Neither site has existing family support services for children with NDI.

Mulago Hospital is the largest in Kampala, Uganda's capital city, taking high-risk pregnancies from across surrounding areas. Children's services include acute admissions, an inpatient malnutrition unit, and outpatients, with a weekly paediatric neurology clinic providing investigation and management of neurological conditions including seizures, and a clinic-based physiotherapy and occupational therapy service for children with cerebral palsy and other NDIs.

For peer review only - http://bmjopen.bmj.com/site/about/guidelines.xhtml

Kiwoko Hospital in Nakaseke District, central Uganda, serves a catchment area of 800,000 people and provides comprehensive medical services, including neonatal inpatient care for >1200 infants per year. The trial implementation partner, Adara Development, has worked in partnership with Kiwoko Hospital since 1998, and the government to improve neonatal health in Nakaseke district. Together they provide HIV services, maternal health services, and community-based healthcare to 44 villages surrounding Kiwoko Hospital.

Participants and recruitment

Participants will be young children with NDI and their caregivers. A SPIRIT diagram showing the planned flow of participants is presented in Figure 1.

Figure 1: Flow of participants

Screening for eligibility

Infants at high-risk of NDI will be identified from i) neonatal admission registers and neonatal follow-up services, ii) local paediatric outpatient services iii) attendance for early child health services following community sensitisation. Sensitisation will include public health announcements on local radio raising awareness of the research and appropriate child development more generally. Caregivers of high-risk infants (survivors of neonatal encephalopathy (NE), prematurity, neonatal septicaemias/meningitis and severe jaundice) will be contacted by phone and invited to attend an appointment when the child is 6-11 completed months old. After informed written consent, they will be screened for NDI by trained study staff using the Malawi Developmental Assessment Tool (MDAT).(12) If two or more items in any MDAT domain are not achieved, the child will be referred for comprehensive neurodevelopmental assessment. If the child fails one item in two or more domains, they will be invited back for an assessment in one month. If the child's MDAT scores are age-appropriate across all domains, advice will be given on play and stimulation, communication, nutrition and immunisations and the child discharged.

Caregivers of infants screening positive on MDAT will be invited to an appointment for written informed consent, and if provided, comprehensive neurodevelopmental assessment by study staff using the Griffiths Mental Developmental Scales (GMDS)(13) and the Hammersmith Infant Neurological Examination (HINE)(14). An overall Developmental Quotient (DQ) will be derived, from the GMDS subscales assessing locomotor, personal-social, hearing/language, eye-hand co-ordination and performance skills.(13) Neuromotor impairment will be further assessed according to the HINE, a standardised paediatric neurological examination and classified by type. We have used both these

For peer review only - http://bmjopen.bmj.com/site/about/guidelines.xhtml

tests extensively in previous studies in Uganda and found them easy to administer in this setting and at this age.(15) The assessments will be conducted in the local language using the standard manual material to ensure internal consistency in assessments technique. Inclusion and exclusion criteria are outlined in Box 1.

Box 1*: Eligibility for inclusion in the RCT*

Inclusion Criteria:

- Infant aged 6-11 completed months
- Moderate-severe NDI defined as a GMDS DQ <70 and/or HINE score <60 (Romeo, 2013)
- Informed written consent by caregiver

Exclusion criteria:

- Infants aged 12 months of age or older
- Infants screening positive for NDI (using MDAT) but not meeting the criteria for moderate-severe NDI on GMDC & HINE assessment
- Conditions requiring prolonged inpatient treatment
- Parents unwilling or unable to attend the full programme
- Main residence outside Nakaseke or Luwero district, and >20km from Mulago Hospital

Baseline characteristics

Infant and caregiver demographic information will be recorded at baseline, including date of birth, age, sex, birth order, parity, antepartum, intrapartum and postpartum history, family and medical history, developmental history, mother's education and occupation, family details including family size, and ages, household incomes, household SES and residence. All outcome measures will also be measured at baseline enabling pre- and post-intervention comparisons.

Randomisation

Infants and their caregivers will be randomised in a 1:1 ratio to either the EIP or SC arm. Randomisation will be stratified by recruitment centre. Randomisation lists indicating a randomisation number and trial arm allocation, will be prepared by the trial statistician using a random number generator in Stata (version 15) prior to the commencement of the study, and stored on a secure, password-protected computer at the Medical Research Council/Uganda Virus Research Institute & LSHTM Uganda Research Unit (MRC/UVRI) by a statistician otherwise not involved in the study. When a participant is eligible for recruitment and consent obtained, study staff will contact the MRC/UVRI statistician who

For peer review only - http://bmjopen.bmj.com/site/about/guidelines.xhtml

will inform the study staff of the study number and trial arm to which the participant is to be allocated. The personnel in charge of the randomisation will not be involved in other study procedures, including assessment of outcomes.

Intervention arm

Box 2. Developing the ABAaNA early intervention programme

In LMICs, services for affected children are often lacking and parental levels of knowledge and understanding about cerebral palsy are often low. To fill this gap, a parent training programme called 'Getting to Know Cerebral Palsy' was developed and launched in partnership between the LSHTM and CBM (Christian Blind Mission) an international disability and development organisation (https://www.ubuntu-hub.org). The programme aims to increase parental knowledge and skills and promotes a participatory learning approach with an emphasis on the empowerment of caregivers across a broad spectrum of impairment for children aged 2-12 years.(17, 18)

Since 2011, the ABAaNA studies ('Abaana', meaning 'children in the local language Luganda) have been examining risk factors for, and outcomes from, neonatal encephalopathy (NE) in Uganda.(15) Studies examining early neurodevelopmental outcomes after NE revealed a high prevalence of NDI with 25% of those affected also having malnutrition from related feeding difficulties.(15) Qualitative work highlighted the stigma and broad-ranging social, emotional and financial impacts on affected families,(3) and the need for an intervention that may improve life chances amongst affected families.

The EIP was developed around the principles of 'Getting to Know Cerebral Palsy' (http://ubuntu-hub.org), and has been adapted for younger children aged 0-2 years following an iterative process following MRC recommendations on development and evaluation of complex interventions,(19); it was supported by a diverse Expert Advisory Group including local parents with children with NDI, Disabled Persons Organisations and experts in early intervention and child development. Core themes running through the programme are summarised in Figure 2. The newly developed programme was piloted amongst 28 families at Mulago Hospital in Kampala in 2015-6 and showed a 25% improvement in family quality of life scores (PedsQL, Family Impact module 2.0) post intervention (verbal communication).

The early intervention programme is a community-based, peer-led group programme with caregivers at a community level, using a participatory approach driven by adult learning theory.(16) The programme manual is freely available to download (https://www.ubuntu-hub.org). Development of the programme is described in Box 2.

For peer review only - http://bmjopen.bmj.com/site/about/guidelines.xhtml

Participating families are encouraged to share experiences through discussion and reflection, prioritise problems and identify solutions together. Facilitators of the group sessions are 'expert parents', themselves parents of children with NDI, who have undergone five days of core training followed by regular supervision, face-to-face mentoring meetings and telephone discussions with existing in-country Master Facilitators (trained therapists in Uganda). Each EIP group involves 6-10 families; groups are selected depending on locality for ease of attendance. The training is divided into ten modules covering understanding disability, positioning and carrying, feeding, mobilising, communication, play, everyday activities, and experiences in the local community (Figure 2, Table 1). Individual module sessions are delivered every 1-2 weeks and last 2-3 hours including time for facilitated discussion; the entire programme is designed to be delivered over six months including at least one home visit conducted by the expert parent facilitator.

Figure 2: Core themes and content of the ABAaNA Early Intervention Programme

Table 1: Description of the programme modules

Module	Content
1. Let's get started	Content and ground rules of the programme<br>Understanding cerebral palsy, additional resources for information<br>Personal stories
2. Know your child	Developmental milestones for young children<br>Determining each child's progress
3. Positioning and carrying	The importance of optimal positioning<br>Practical skills regarding optimal positioning
4. Eating and drinking	Feeding challenges for children with neurodevelopmental impairment<br>Practical skills for addressing feeding challenges
5. Learning to move	Understanding different types of movement<br>Practical skills for assisting learning to move
6. Communicating	The importance of communication<br>Practical advice to encourage their child to communicate
7. Play and early stimulation	The importance of early stimulation and play for children to develop<br>Challenges of inclusion in play with the family and community<br>Creation of simple toys<br>How parents/ caregivers can encourage their child to play
8. Everyday activities	Using everyday activities to promote child development<br>Management of seizures<br>Review of previous sessions
9. Our community	Community resources available<br>Discussion around barriers to inclusion, addressing stigma and discrimination<br>Understanding disability rights<br>Thoughts and feelings of the caregiver<br>Members of community invited to attend this session
10. Next steps	Planning to facilitate their own group<br>Reflection on learning points

For peer review only - http://bmjopen.bmj.com/site/about/guidelines.xhtml

	Endpoint data collection

Fidelity & Adherence to the intervention

EIP facilitators will receive a 5-day training programme delivered by two Master Facilitators, which includes facilitation skills, knowledge transfer on the core contents of the EIP manual and translation of knowledge to practice through simulated sessions with families and children with NDI. All trial intervention groups will be co-facilitated by a Master Facilitator providing supportive supervision to new facilitators. After each modular group meeting, a short-facilitated feedback session will be conducted, and the content of the module delivered will be recorded. Attendance of individual caregivers and children at the group sessions will be recorded. Facilitators will emphasize to caregivers the importance of attending all sessions, with phone calls prior to each session to promote adherence. If missed, a catch-up session may be offered before the next module.

Standard care arm

SC refers to care that is currently available in established local services. In both sites this includes referral to physiotherapy, seizure management and nutritional support. Information on access to local medical, therapy and family services will be collected. Families in the SC arm will be offered delayed entry into the EIP after completing the 18-month assessment. Contamination of the SC arm by exposure of SC families to intervention will be monitored and reported.

Outcomes

Participants in both arms will be assessed by study staff masked to trial allocation at two time points; at age 12-17 months (which corresponds to completion of the EIP in the intervention arm, 6 months after recruitment) and again at age 18-23 months (12 months after recruitment). (Figure 1). Caregivers will be phoned a week before the follow-up assessments to arrange a time for interview. Assessments will be primarily conducted in the study-site clinics. Where caregivers cannot be contacted by phone or are unable to attend the clinic, a community visit will be arranged, and assessments completed at home. Outcome assessments will be conducted by Mulago assessors for children recruited at Kiwoko, and vice versa to ensure assessors are blind to allocation arm. Two assessors will independently assess a small proportion of the children and Inter-rater reliability will be calculated.

Primary outcome measures

The primary outcomes of the study will be:

For peer review only - http://bmjopen.bmj.com/site/about/guidelines.xhtml

1. *Feasibility of participant recruitment and randomisation* as assessed by the total number recruited and randomised to each arm. Qualitative tools will also be used to capture information on feasibility.
2. *Acceptability of the EIP amongst caregivers and health care workers* as assessed by the protocol violation rate (e.g. participants in the intervention arm being treated as if they were in the control arm or vice versa) at programme completion, and by the number of programme sessions attended between baseline and programme completion. Qualitative tools will also be used to capture information on acceptability.
3. *Preliminary evidence of impact on Family Quality of Life* as assessed using the scored Pediatric Quality of Life Family Impact module (PedsQL),(20) The PedsQL comprises 36 items scored on a 0-4 Likert scale and linearly transformed to a 0-100 scale, with higher scores indicating a better QoL. It will be translated into the local language Luganda and administered as a standardised structured interview by trained study staff.

Other outcomes of interest

1. *Child motor functioning* as assessed by the mobility score of the Pediatric Evaluation Disability Inventory (PEDI).(21) The PEDI is a standardised test designed to identify and describe functional impairment and monitor progress. Normative scaled scores are obtained for children ≥6 months to provide age-related expectations of ability.
2. *Child cognitive function* as assessed by the GMDS.(13)
3. *Child growth, health and well-being* assessed using weight, height and head circumference measured according to standardised protocol. Occipito-frontal head circumference (OFC, paper tape measure), weight, (SECA336 electronic scales, Hamburg, Germany) and height will be taken by study staff using standardised procedures. Haemoglobin will be determined on a finger prick sample using HemoCue Hb 201 (HemoCue AB, Angelholm, Sweden). A structured maternal interview in Luganda will report on caregivers concerns regarding health, growth and development of their child and episodes of illness including seizures and other neurological problems, feeding difficulties, chest infections, and treatment for malnutrition.
4. *Caregiver psychological distress* assessed using the Self-Referral Questionnaire (SRQ) and the Parenting Stress index (PSI).(22) The SRQ consists of 20 items each scored 0 (symptom absent) or 1 (symptom present) giving a total out of 20. The PSI is a 120-item inventory measuring the magnitude of caregiver stress attributable to parent-child relationship (Total Stress Scale), and situational/demographics factors outside the parent-child relationship (Life Stress Scale). These tools will be translated into Luganda.

For peer review only - http://bmjopen.bmj.com/site/about/guidelines.xhtml

5. *Caregiver-child attachment* assessed using the Maternal Infant Responsiveness Instrument (MIRI); a 22-item scale designed to measure the parent's feelings about their infant and an appraisal of the infant's responses.(23)
6. *Quality of the home environment* assessed using the Infant Toddler-Home Observation for the Measurement of the Environment (IT-HOME). This comprises 45 items, based on observation and/or interview, assessing the physical environment of the home and the child's interaction within it.(24)
7. *Cost of illness and protective effectiveness* will be assessed (separate protocol, Katumba et al)

Qualitative methods

IDIs will be conducted with five randomly selected caregivers from each arm at each site. FGDs will be conducted with caregivers, at baseline, 6 months post-recruitment and again 6 months later in both the intervention and SC arms. Amongst intervention arm families, qualitative techniques will be used to capture information on the feasibility, acceptability and impact of the EIP intervention using qualitative tools including focus group discussions (FGDs), in-depth-interviews (IDIs) and observation.

We will describe the experiences of children and caregivers relating to the intervention received including the impact of the disability, parental confidence level, inclusion in community life and experience of stigma and discrimination. We will examine changes in these domains over the follow-up period and explore attributions of change. In addition, we will perform social mapping of parent networks and group discussions with staff on their perspectives and experiences of using the EIP. The themes guiding our analysis will be drawn not only from objectives of the trial but also from the data, should additional areas of interest emerge during interviews and discussions.

The interviews will be conducted by social scientists who have experience in qualitative research.

Data management and access

Data collected in the clinic or at field visits will be entered on standard clinical record forms (CRFs). Clinical data will be recorded under a unique study ID number. Completed CRFs will be checked by and double-entered into a trial-specific MS Access database. Data from both IDIs and FGDs will be collected in the form of audiotapes, transcripts and field notes. All data entry and data management will be overseen by a statistician/data manager at the MRC/UVRI Unit. Data will be maintained on the host institution server and backed-up following standardised operating procedures. Paper CRFs will be stored in lockable filing cabinets at the sites. Access to these data during the trial will be restricted to essential personnel (the PIs, site co-investigators, medical research officers, and data clerks).

For peer review only - http://bmjopen.bmj.com/site/about/guidelines.xhtml

For peer review only

Confidentiality

All research-team members will receive training in confidentiality. Data will be stored without personal identifiers, except where names must be included to ensure identification of the correct participants for procedures. All data will be stored on password-protected computers, accessible only to research team members.

Sample size

The trial will recruit 126 children and their caregivers; 63 per arm. Allowing for a 20% dropout rate, this sample size will give 90% power to detect a minimal relative difference of 20% on PedQL Family Impact score between the intervention and control arms, at 5% significance level, assuming a mean PedQL score of 65 in the standard care arm and SD of 20 in both arms. Assumptions are based on data from the pilot study showing a mean caregiver PedQL score for families before the intervention of 64.9 (standard deviation (SD) 19.6) and mean score of 78.9 for families after receiving the intervention (SD 17.5).

Statistical analysis

The first primary outcome, feasibility of participant recruitment and randomisation, will be assessed by the total number recruited and randomised to each arm. Recruitment and randomisation feasibility will be demonstrated if the target sample size of 126 is achieved. Data on participants screened, eligible and randomised will be displayed in a CONSORT flowchart. Descriptive statistics (frequencies, means, medians, standard deviations and interquartile ranges) will be used to describe the sample at baseline, by trial arm

The second primary outcome, acceptability, will be assessed quantitatively by (a) calculating the protocol violation rate and (b) summarising the number of programme sessions attended between baseline and programme completion for those in the intervention arm. Protocol violation rate will be calculated as the number of participants for whom one or more protocol violations occur divided by the total number of participants, and will be presented both overall, and by trial arm. For participants in the EIP trial arm, the overall number of modules attended by each participant will be tabulated. Acceptability on the basis of number of programme sessions will be defined as attendance of at least 6 modules.

For the third primary outcome and secondary outcomes, analyses will compare outcomes between intervention and control arms at the end of the programme, when the participants will be aged 12-17 months, and again 6 months later. Analysis will be on an intention-to-treat basis and missing data

For peer review only - http://bmjopen.bmj.com/site/about/guidelines.xhtml

will not be imputed. Data for each outcome measure will be summarised by trial arm, using proportions for binary outcomes and means or medians for quantitative outcomes, depending on normality of the distribution. Differences in means/proportions between trial arms together with 95% confidence intervals will be calculated. We do not plan any formal statistical tests due to the preliminary nature of the trial; instead confidence intervals will provide a possible range of effect sizes. Regression models (linear regression for continuous outcomes, logistic regression for binary outcomes) will be used to adjust comparisons for baseline measures of the outcomes, which were collected at enrolment into the trial, in order to improve precision of effectiveness estimates. For skewed continuous outcomes, data will be normalised before analysis using suitable transformations or quantile regression will be considered. No subgroup analyses are planned.

Qualitative data will be analysed using a thematic framework approach. Themes will be based on the study objectives and those emerging from the data. Social scientists (two people) will agree the coding frame and undertake analysis collaboratively to ensure agreement on the coding approach. Thematic summaries will be developed and shared with the wider team for discussion.

Trial management, data monitoring and reporting of adverse events

The TSC (25) will oversee progress of the study towards its objectives, review relevant information from other sources (e.g. other related trials) and receive reports from the Data and Safety Monitoring Board (DSMB). All adverse events, whether related to the intervention or not, will be noted and reported. A Data Monitoring and Safety Committee (DSMC) has been established independent of the investigators and the TSC but reporting to the TSC and the sponsor. The DSMB includes an expert on global child heath, a senior statistician, and a senior academic working in newborn and early child health research in Uganda, independent of the investigators. The DSMB will have access to all data on request. Resulting from the initial meeting of the DSMB on 28th June 2017, no formal stopping rules will be applied.

Children with NDI and particularly those with seizure disorders and difficulties with swallowing, are at increased mortality risk. All adverse events, whether related to the intervention or not, will be investigated and reported according to the UVRI Research Ethics Committee in accordance with GCP requirements. All deaths, hospitalisations and other serious adverse effects will be reported to the relevant ethics committee irrespective of whether the death or event is related to disease progression or not. Trial data monitoring will be conducted by an internal independent monitor at initiation, 6 months into data collection, again after one year and end of data collection.

For peer review only - http://bmjopen.bmj.com/site/about/guidelines.xhtml

For peer review only

Participant and public involvement

The intervention, study design and conduct, were developed directly from the engagement of caregivers and programme facilitators ('expert parents') with a parent representative on the TSC. The priorities and experiences of caregivers identified during facilitated group discussions at a key-stakeholders meeting (June 2017) contributed to the development of our research question and outcome measures. Plans to communicate findings to participants and the wider community will involve caregivers, through formal discussions with the TSC.

Ethics & Dissemination

Ethics

The protocol has been approved by the Research and Ethics committee of the UVRI, Mulago Hospital and Kiwoko Hospitals, the Uganda National Council for Science and Technology, the Uganda President's Office, and the ethics committee of the LSHTM. Information sheets will be available in English and Luganda, the main local language. Parents will be provided with an oral and written explanation of the study by Ugandan study staff to ensure that information is accessible to those with lower levels of literacy. Witnessed consent using a thumb print will be available to parents/guardians who are non-literate. Reimbursement for the cost of transport will be provided to caregivers on attendance at the screening and recruitment visits.

All recruited children will receive SC at the study sites. This will include referral to local services for seizure management and physiotherapy where available. To date the benefits of the proposed EIP have not been proven and may have a negative effect if children are incorrectly classified as having NDI and placed in the programme. Children and their caregivers in the control arm will receive delayed entry into the programme for older children ('Getting to Know Cerebral Palsy') at 18-23 months at the time of their final study assessments.

Dissemination

Our programme has strong links with partnership organisations working in Maternal and Child Health programming including Adara Development, Kiwoko Hospital, Nakaseke District Health Office and other collaborating institutions. Research findings will be disseminated to the Ministry of Health, to inform local and national health policies. Regional level stakeholders including the Nakaseke District Health Office and heads of regional health and social services, will be engaged to support staff recruitment, contributing to the sustainability of the innovation at local and district level. Meetings for key-stakeholders, including local NGOs working in child disability will be held twice

For peer review only - http://bmjopen.bmj.com/site/about/guidelines.xhtml

during the project period to promote buy-in, facilitate fast-cycle learning, disseminate study findings and ultimately promote sustainability of the programme. Global learning will be facilitated through our existing online community-of-practice spanning 70 countries and >300 members. Communications support staff at MRC/UVRI, LSHTM and Adara Development will facilitate dissemination of information through appropriate media outlets, the web and social media.

Study findings will be published through Open Access peer-reviewed journals, presentations at local, national and international conferences and to the local community through community meetings. Written reports will be submitted to UVRI REC and reported to the trial registry. Data will be made available upon request.

Authors contributions

The study was conceived and designed by CT with substantial contribution from MN, DL, JN, EW, CM, JS, KK and FC. Research methodology was developed by MN, CN, MZ, JN, BM, DK, SS, RN, MS, FC, MMB, AM and CT. The first version of the paper was written by CT, SS & MN. All authors contributed to the final version of the manuscript.

Acknowledgements

The authors would like to thank the members of the ABAaNA Early Intervention Programme Expert Advisory Committee who supported the development of the programme and the implementation partners Kiwoko Hospital, Adara Development and Mulago University Hospital. They would like to acknowledge the contribution of Ellipsis for formatting the programme manual and thank Joanna Lawn for her contribution to manual illustrations. The authors would like to thank the funders, Grand Challenges Canada, Saving Brains and the Research Foundation of Cerebral Palsy Alliance Australia.

Funding statement

This work is supported by Saving Brains, Grand Challenges Canada grant number 1707-08687. The economic evaluation is supported by a Project Grant, awarded by the Research Foundation, Cerebral Palsy Alliance (PG02917).

The Trial sponsor is the MRC/UVRI and LSHTM Uganda Research Unit, Entebbe; Contact name: Prof Pontiano Kaleebu (Director); Address: MRC/UVRI & LSHTM Uganda Research Unit, Plot 51-59 Nakiwogo Road, P.O. Box 49 Entebbe, UGANDA Tel: +256 (0) 417 704000; mrc@mrcuganda.org

For peer review only - http://bmjopen.bmj.com/site/about/guidelines.xhtml

The funder had no role in the research design and will not have any role in the execution, analyses, interpretation of the data, or decision to submit results.

Data Sharing Statement

This is a study protocol and therefore no unpublished data are yet available. Data collected during the course of the trial will be made available on request, after the main study findings have been accepted for publication.

Competing interest statement

We declare no competing interests.

References

1. WHO U. Survive and Thrive: Transforming Care for Every Small and Sick Newborn. Key Findings 2018 [Available from: https://www.unicef.org/every-child-alive/Survive-and-Thrive_KEY_FINDINGS_FINAL.pdf.
2. Olusanya BO, Davis AC, Wertlieb D, Boo N-Y, Nair MKC, Halpern R, et al. Developmental disabilities among children younger than 5 years in 195 countries and territories, 1990–2016: a systematic analysis for the Global Burden of Disease Study 2016. The Lancet Global Health. 2018;6(10):e1100-e21.
3. Nakamanya S, Siu GE, Lassman R, Seeley J, Tann CJ. Maternal experiences of caring for an infant with neurological impairment after neonatal encephalopathy in Uganda: a qualitative study. Disability and rehabilitation. 2015;37(16):1470-6.
4. Morgan C, Darrah J, Gordon AM, Harbourne R, Spittle A, Johnson R, et al. Effectiveness of motor interventions in infants with cerebral palsy: a systematic review. Developmental medicine and child neurology. 2016;58(9):900-9.
5. Wallander JL, McClure E, Biasini F, Goudar SS, Pasha O, Chomba E, et al. Brain research to ameliorate impaired neurodevelopment--home-based intervention trial (BRAIN-HIT). BMC Pediatr. 2010;10:27.
6. Palmer FB, Shapiro BK, Wachtel RC, Allen MC, Hiller JE, Harryman SE, et al. The effects of physical therapy on cerebral palsy. A controlled trial in infants with spastic diplegia. N Engl J Med. 1988;318(13):803-8.
7. Bao X, Sun S, Wei S. Early intervention promotes intellectual development of premature infants: a preliminary report. Early Intervention of Premature Infants Cooperative Research Group. Chin Med J (Engl). 1999;112(6):520-3.
8. Gardner JM, Walker SP, Powell CA, Grantham-McGregor S. A randomized controlled trial of a home-visiting intervention on cognition and behavior in term low birth weight infants. J Pediatr. 2003;143(5):634-9.
9. Walker SP, Chang SM, Powell CA, Grantham-McGregor SM. Psychosocial intervention improves the development of term low-birth-weight infants. J Nutr. 2004;134(6):1417-23.
10. Carlo WA, Goudar SS, Pasha O, Chomba E, Wallander JL, Biasini FJ, et al. Randomized trial of early developmental intervention on outcomes in children after birth asphyxia in developing countries. J Pediatr. 2013;162(4):705-12 e3.
11. Benfer KA, Novak I, Morgan C, Whittingham K, Khan NZ, Ware RS, et al. Community-based parent-delivered early detection and intervention programme for infants at high risk of cerebral palsy in a low-resource country (Learning through Everyday Activities with Parents (LEAP-CP): protocol for a randomised controlled trial. BMJ open. 2018;8(6):e021186.

For peer review only - http://bmjopen.bmj.com/site/about/guidelines.xhtml

For peer review only

12. Gladstone M, Lancaster GA, Umar E, Nyirenda M, Kayira E, van den Broek NR, et al. The Malawi Developmental Assessment Tool (MDAT): the creation, validation, and reliability of a tool to assess child development in rural African settings. PLoS Med. 2010;7(5):e1000273.
13. Griffiths R, Huntley M. The Griffiths mental development scales : from birth to two years : manual. Henley on Thames: ARICD; 1996.
14. Haataja L, Mercuri E, Regev R, Cowan F, Rutherford M, Dubowitz V, et al. Optimality score for the neurologic examination of the infant at 12 and 18 months of age. J Pediatr. 1999;135(2 Pt 1):153-61.
15. Tann CJ, Webb EL, Lassman R, Ssekyewa J, Sewegaba M, Musoke M, et al. Early Childhood Outcomes After Neonatal Encephalopathy in Uganda: A Cohort Study. EClinicalMedicine. 2018;6:26-35.
16. Knowles M. The Adult Learning Theory - Andragogy 1984 [Available from: https://elearningindustry.com/the-adult-learning-theory-andragogy-of-malcolm-knowles.
17. Zuurmond M, O'Banion D, Gladstone M, Carsamar S, Kerac M, Baltussen M, et al. Evaluating the impact of a community-based parent training programme for children with cerebral palsy in Ghana. PloS one. 2018;13(9):e0202096.
18. Zuurmond M, Nyante G, Baltussen M, Seeley J, Abanga J, Shakespeare T, et al. A support programme for caregivers of children with disabilities in Ghana: Understanding the impact on the wellbeing of caregivers. Child Care Health Dev. 2019;45(1):45-53.
19. Campbell M, Fitzpatrick R, Haines A, Kinmonth AL, Sandercock P, Spiegelhalter D, et al. Framework for design and evaluation of complex interventions to improve health. BMJ. 2000;321(7262):694-6.
20. Varni JW, Burwinkle TM, Seid M, Skarr D. The PedsQL 4.0 as a pediatric population health measure: feasibility, reliability, and validity. Ambul Pediatr. 2003;3(6):329-41.
21. Haley SM, Coster WJ, Ludlow LH, Haltiwanger JT, Andrellos PJ. Pediatric Evaluation of Disability Inventory. Pediatric Physical Therapy Pediatric Physical Therapy. 1994;6(1):42.
22. Abidin RR. Parenting Stress Index. Lutz, FL: PAR; 2012.
23. Amankwaa L. The Maternal Infant Responsiveness Instrument 2002 [Available from: https://miriinstrument.wordpress.com/.
24. Caldwell BM, Bradley RH, University of Arkansas at Little R, Center for Child D, Education. Administration manual : HOME observation for measurement of the environment. Little Rock, Ark.: University of Arkansas at Little Rock; 1984.
25. MRC. Guidelines for good clinical practice in clinical trials 1998 [Available from: https://mrc.ukri.org/documents/pdf/good-clinical-practice-in-clinical-trials/.

For peer review only

For peer review only - http://bmjopen.bmj.com/site/about/guidelines.xhtml

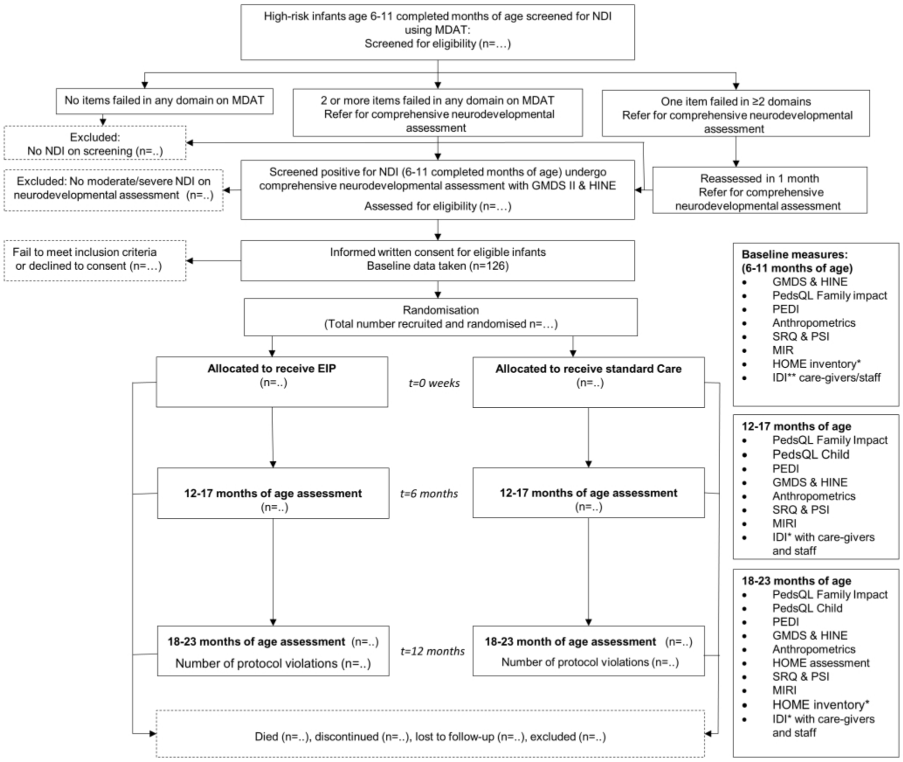

*In-depth interviews (IDI) with caregivers on impact of disability, confidence level of the parents, level of participation in family and community life and experience of stigma/discrimination. MDAT=Malawi Developmental Assessment Tool, PedQL=Pediatric Quality of Life tool, PEDI=Pediatric Evaluation Disability Inventory GMDS=Griffiths Mental Developmental Scales, HINE=Hammersmith Infant Neurological Examination, HOME=Home Observation for the Measurement of the Environment, SRQ=Self-Referral Questionnaire, PSI=Parent Stress Index MIRI=Maternal Infant Responsiveness Inventory

309x339mm (300 x 300 DPI)

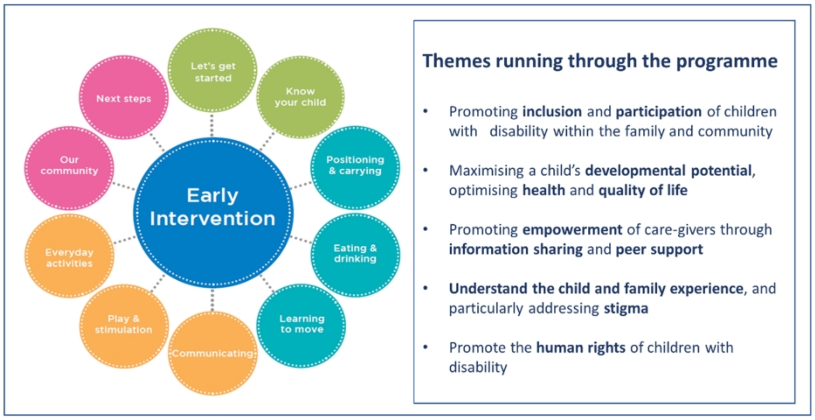

Figure 2: Core themes and content of the ABAaNA Early Intervention Programme

338x190mm (300 x 300 DPI)

SPIRIT

STANDARD PROTOCOL ITEMS: RECOMMENDATIONS FOR INTERVENTIONAL TRIALS

SPIRIT 2013 Checklist: Recommended items to address in a clinical trial protocol and related documents*

Section/item	Item No	Description	Addressed on page number
**Administrative information**			
Title	1	Descriptive title identifying the study design, population, interventions, and, if applicable, trial acronym	_______1_______
Trial registration	2a	Trial identifier and registry name. If not yet registered, name of intended registry	_______2_______
	2b	All items from the World Health Organization Trial Registration Data Set	_______N/A_____
Protocol version	3	Date and version identifier	_______2______
Funding	4	Sources and types of financial, material, and other support	_______16____
Roles and responsibilities	5a	Names, affiliations, and roles of protocol contributors	_____1 &______
	5b	Name and contact information for the trial sponsor	______1________
	5c	Role of study sponsor and funders, if any, in study design; collection, management, analysis, and interpretation of data; writing of the report; and the decision to submit the report for publication, including whether they will have ultimate authority over any of these activities	_______16____
	5d	Composition, roles, and responsibilities of the coordinating centre, steering committee, endpoint adjudication committee, data management team, and other individuals or groups overseeing the trial, if applicable (see Item 21a for data monitoring committee)	_____13/14____

For peer review only

For peer review only - http://bmjopen.bmj.com/site/about/guidelines.xhtml

Introduction

Background and rationale	6a	Description of research question and justification for undertaking the trial, including summary of relevant studies (published and unpublished) examining benefits and harms for each intervention	______4______
	6b	Explanation for choice of comparators	______4___
Objectives	7	Specific objectives or hypotheses	______5______
Trial design	8	Description of trial design including type of trial (eg, parallel group, crossover, factorial, single group), allocation ratio, and framework (eg, superiority, equivalence, noninferiority, exploratory)	_____4-5______

Methods: Participants, interventions, and outcomes

Study setting	9	Description of study settings (eg, community clinic, academic hospital) and list of countries where data will be collected. Reference to where list of study sites can be obtained	_____5-6_____
Eligibility criteria	10	Inclusion and exclusion criteria for participants. If applicable, eligibility criteria for study centres and individuals who will perform the interventions (eg, surgeons, psychotherapists)	_____7 (Box 1)_
Interventions	11a	Interventions for each group with sufficient detail to allow replication, including how and when they will be administered	____8-10_____
	11b	Criteria for discontinuing or modifying allocated interventions for a given trial participant (eg, drug dose change in response to harms, participant request, or improving/worsening disease)	____N/A_____
	11c	Strategies to improve adherence to intervention protocols, and any procedures for monitoring adherence (eg, drug tablet return, laboratory tests)	____10_______
	11d	Relevant concomitant care and interventions that are permitted or prohibited during the trial	____10_______
Outcomes	12	Primary, secondary, and other outcomes, including the specific measurement variable (eg, systolic blood pressure), analysis metric (eg, change from baseline, final value, time to event), method of aggregation (eg, median, proportion), and time point for each outcome. Explanation of the clinical relevance of chosen efficacy and harm outcomes is strongly recommended	____10-11____
Participant timeline	13	Time schedule of enrolment, interventions (including any run-ins and washouts), assessments, and visits for participants. A schematic diagram is highly recommended (see Figure)	__10 &Figure 2

For peer review only - http://bmjopen.bmj.com/site/about/guidelines.xhtml

Sample size	14	Estimated number of participants needed to achieve study objectives and how it was determined, including clinical and statistical assumptions supporting any sample size calculations	___12-13_____
Recruitment	15	Strategies for achieving adequate participant enrolment to reach target sample size	___6-7______

Methods: Assignment of interventions (for controlled trials)

Allocation:

Sequence generation	16a	Method of generating the allocation sequence (eg, computer-generated random numbers), and list of any factors for stratification. To reduce predictability of a random sequence, details of any planned restriction (eg, blocking) should be provided in a separate document that is unavailable to those who enrol participants or assign interventions	____7-8______
Allocation concealment mechanism	16b	Mechanism of implementing the allocation sequence (eg, central telephone; sequentially numbered, opaque, sealed envelopes), describing any steps to conceal the sequence until interventions are assigned	____7-8_____
Implementation	16c	Who will generate the allocation sequence, who will enrol participants, and who will assign participants to interventions	_____7-8______
Blinding (masking)	17a	Who will be blinded after assignment to interventions (eg, trial participants, care providers, outcome assessors, data analysts), and how	___2,5,10______
	17b	If blinded, circumstances under which unblinding is permissible, and procedure for revealing a participant's allocated intervention during the trial	____13-14______

Methods: Data collection, management, and analysis

Data collection methods	18a	Plans for assessment and collection of outcome, baseline, and other trial data, including any related processes to promote data quality (eg, duplicate measurements, training of assessors) and a description of study instruments (eg, questionnaires, laboratory tests) along with their reliability and validity, if known. Reference to where data collection forms can be found, if not in the protocol	____6-11______
	18b	Plans to promote participant retention and complete follow-up, including list of any outcome data to be collected for participants who discontinue or deviate from intervention protocols	____10______

Data management	19	Plans for data entry, coding, security, and storage, including any related processes to promote data quality (eg, double data entry; range checks for data values). Reference to where details of data management procedures can be found, if not in the protocol	_____12______
Statistical methods	20a	Statistical methods for analysing primary and secondary outcomes. Reference to where other details of the statistical analysis plan can be found, if not in the protocol	_____13_______
	20b	Methods for any additional analyses (eg, subgroup and adjusted analyses)	_______N/A_____
	20c	Definition of analysis population relating to protocol non-adherence (eg, as randomised analysis), and any statistical methods to handle missing data (eg, multiple imputation)	_______13_______
**Methods: Monitoring**			
Data monitoring	21a	Composition of data monitoring committee (DMC); summary of its role and reporting structure; statement of whether it is independent from the sponsor and competing interests; and reference to where further details about its charter can be found, if not in the protocol. Alternatively, an explanation of why a DMC is not needed	_____13-14____
	21b	Description of any interim analyses and stopping guidelines, including who will have access to these interim results and make the final decision to terminate the trial	____13-14_____
Harms	22	Plans for collecting, assessing, reporting, and managing solicited and spontaneously reported adverse events and other unintended effects of trial interventions or trial conduct	_____14______
Auditing	23	Frequency and procedures for auditing trial conduct, if any, and whether the process will be independent from investigators and the sponsor	____14_______
**Ethics and dissemination**			
Research ethics approval	24	Plans for seeking research ethics committee/institutional review board (REC/IRB) approval	____14_______
Protocol amendments	25	Plans for communicating important protocol modifications (eg, changes to eligibility criteria, outcomes, analyses) to relevant parties (eg, investigators, REC/IRBs, trial participants, trial registries, journals, regulators)	_____15______

For peer review only - http://bmjopen.bmj.com/site/about/guidelines.xhtml

Consent or assent	26a	Who will obtain informed consent or assent from potential trial participants or authorised surrogates, and how (see Item 32)	_____14______
	26b	Additional consent provisions for collection and use of participant data and biological specimens in ancillary studies, if applicable	_____N/A________
Confidentiality	27	How personal information about potential and enrolled participants will be collected, shared, and maintained in order to protect confidentiality before, during, and after the trial	_____12________
Declaration of interests	28	Financial and other competing interests for principal investigators for the overall trial and each study site	_____16________
Access to data	29	Statement of who will have access to the final trial dataset, and disclosure of contractual agreements that limit such access for investigators	_____15________
Ancillary and post-trial care	30	Provisions, if any, for ancillary and post-trial care, and for compensation to those who suffer harm from trial participation	______14-15____
Dissemination policy	31a	Plans for investigators and sponsor to communicate trial results to participants, healthcare professionals, the public, and other relevant groups (eg, via publication, reporting in results databases, or other data sharing arrangements), including any publication restrictions	_____15________
	31b	Authorship eligibility guidelines and any intended use of professional writers	_____16________
	31c	Plans, if any, for granting public access to the full protocol, participant-level dataset, and statistical code	____15_________
**Appendices**			
Informed consent materials	32	Model consent form and other related documentation given to participants and authorised surrogates	__online______
Biological specimens	33	Plans for collection, laboratory evaluation, and storage of biological specimens for genetic or molecular analysis in the current trial and for future use in ancillary studies, if applicable	_____N/A________

*It is strongly recommended that this checklist be read in conjunction with the SPIRIT 2013 Explanation & Elaboration for important clarification on the items. Amendments to the protocol should be tracked and dated. The SPIRIT checklist is copyrighted by the SPIRIT Group under the Creative Commons "Attribution-NonCommercial-NoDerivs 3.0 Unported" license.
